# Targeting the NOTCH2/ADAM10/TCF7L2 Axis‐Mediated Transcriptional Regulation of Wnt Pathway Suppresses Tumor Growth and Enhances Chemosensitivity in Colorectal Cancer

**DOI:** 10.1002/advs.202405758

**Published:** 2024-11-27

**Authors:** Zhen Xiang, Yiwei Wang, Xiao Ma, Shuzheng Song, Yuanqiao He, Jiamin Zhou, Longhai Feng, Su Yang, Yibin Wu, Bingran Yu, Guangkai Xia, Weiqi Xu, Yiming Zhao, Lu Wang

**Affiliations:** ^1^ Department of Hepatic Surgery Fudan University Shanghai Cancer Center 270 Dong‐An Road Shanghai 200032 China; ^2^ Department of general surgery Shanghai Jiao Tong University Affiliated Sixth People's Hospital 600 Yishan Rd Shanghai 200233 P. R. China; ^3^ Fudan University Shanghai Cancer Center 270 Dong‐An Road Shanghai 200032 P. R. China; ^4^ Department of Colorectal Surgery Department of General Surgery Shanghai East Hospital Tongji University School of Medicine 150 Jimo Road Shanghai 200120 P. R. China; ^5^ Center of Laboratory Animal Science Nanchang University No.999, Xuefu Road Nanchang 330031 P. R. China; ^6^ Department of Colorectal Surgery The Cancer Hospital of the University of Chinese Academy of Sciences (Zhejiang Cancer Hospital) 1 Banshan East Road Hangzhou 310022 P. R. China; ^7^ Department of Thoracic Surgery Ruijin Hospital Shanghai Jiaotong University School of Medicine 197 Ruijin 2nd Road Shanghai 200025 P. R. China

**Keywords:** ADAM10, colorectal cancer, liver metastasis, NOTCH2, organoid, TCF7L2, Wnt pathway

## Abstract

Wnt/β‐catenin/transcription factor (TCF) transcriptional activity plays an integral role in colorectal cancer (CRC) carcinogenesis. However, to date, no drugs targeting this pathway are used in clinical practice owing to the undesirable and serious side effects. In this study, it is found that the transcriptional regulation of Wnt pathway is activated and associated with liver metastasis in CRC. Through high‐throughput screening of 24 inhibitors on 12 CRC and three colorectal organoids in this organoid living biobank, adavivint is found to exhibit anti‐tumor activity and low toxicity in colorectal organoids, independent of the canonical Wnt/β‐catenin signaling. Mechanistically, ADAM10 is screened as a target of adavivint to specifically regulate the protein expression of NOTCH2, which mediates the transcriptional regulation of the Wnt pathway. NOTCH2 not directly interact with TCF7‐like 2 (TCF7L2), a key downstream transcriptional factor of canonical Wnt/β‐catenin signaling, but directly activated the transcription of TCF7L2 and Wnt target genes, such as *MYC*, *JUN and CCND1/2*. Furthermore, use of adavivint or blockage of ADAM10/NOTCH2/TCF7L2 signaling enhances the chemosensitivity of CRC cells. Overall, this study provides a promising candidate for the development of small‐molecule inhibitors and reveals a potential therapeutic target for CRC.

## Graphical abstract

Transcriptional regulation of Wnt pathway is activated and associated with liver metastasis in CRC. By high‐throughput screening of inhibitors with CRC organoids, adavivint is found to suppress tumor growth and enhance chemotherapy sensitivity of CRC by targeting transcriptional regulation of Wnt pathway. Further exploring the action mechanisms of adavivint identifies ADAM10/NOTCH2/TCF7L2 signaling as a potential therapeutic target for CRC.

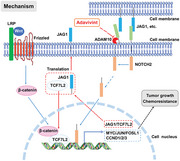

## 1 Introduction

Colorectal cancer (CRC) is among the most prevalent malignancies and a primary contributor to cancer‐related mortality.^[^ ^1^ ^]^ Approximately 50% of patients diagnosed with CRC develop liver metastases^[^ ^2^ ^]^ with a 5‐year survival rate of less than 10%.^[^ ^3^ ^]^ Surgical intervention, systemic chemotherapy, and targeted therapies, such as those involving cetuximab and bevacizumab, are the primary treatment options for CRC liver metastases. Owing to its considerable heterogeneity and complexity, CRC exhibits varied responses to chemotherapy and targeted therapies, and acquired drug resistance during treatment significantly impedes therapeutic efficacy. Improving the prognosis of patients with chemotherapy‐resistant metastatic CRC remains a challenge. Therefore, novel therapeutic targets are urgently needed to overcome drug resistance in CRC treatment.

CRC is caused by the accumulation of multiple gene mutations, such as *APC*, *TP53*, and *KRAS*.^[^ ^4^, ^5^ ^]^ Specifically, *APC* mutation‐mediated overactivation of the Wnt signaling pathway is a key driver of CRC carcinogenesis, with approximately 80% of CRC cases harboring *APC* mutations.^[^ ^4^, ^6^ ^]^ Mechanistically, *APC* mutations accelerate the nuclear accumulation of β‐catenin, leading to its binding with transcription factor (TCF)/lymphoid enhancer factor (LEF) and activation of transcription for downstream target genes of the classical Wnt pathway, including MYC, JUN, etc.^[^ ^6^, ^7^, ^8^, ^9^ ^]^ Additionally, activation of Wnt pathway also promotes CRC progression by influencing the tumor microenvironment, cell proliferation, apoptosis, autophagy, metabolism, invasion, and migration.^[^ ^10^, ^11^ ^]^ Furthermore, Wnt pathway can crosstalk with the Notch and Sonic Hedgehog pathways, presenting significant implications for cancer therapy.^[^ ^12^, ^13^ ^]^ Although the Wnt pathway plays a crucial role in tumorigenesis and tumor development, an increasing number of studies have reported that it is regulated by other pathways, including the KRAS, phosphoinositide 3‐kinase–Akt, and Notch pathways.^[^ ^14^ ^]^ These findings indicate that targeting crucial upstream regulatory components may be a desirable approach for treating Wnt pathway‐active CRC.

Despite the critical role of the Wnt signaling pathway in carcinogenesis and tumor development, there is currently no approved drug targeting this pathway.^[^ ^15^, ^16^ ^]^ Several inhibitors targeting the canonical Wnt/β‐catenin pathway, including porcupine O‐acyltransferase (PORCN) inhibitors and anti‐R‐spondin (RSPO) antibodies, are currently undergoing clinical trials.^[^ ^15^ ^]^ The Wnt signaling pathway also mediates resistance to conventional chemotherapy and radiotherapy.^[^ ^10^ ^]^ Inhibition of Wnt ligand secretion by PORCN inhibitors or RSPO3 blockade leads to rapid and sustained tumor regression.^[^ ^17^, ^18^, ^19^ ^]^ The canonical Wnt/β‐catenin pathway regulates cell proliferation, development, self‐renewal, and differentiation. However, Wnt pathway inhibitors also exerts on‐target adverse effects.^[^ ^16^ ^]^ For instance, blocking the Wnt pathway using tankyrase inhibitors induces severe gut toxicity in mice used for cancer treatment, thereby limiting the application of tankyrase inhibitors for human patients ^[^ ^17^, ^19^ ^]^. Although PORCN inhibitors do not exhibit significant side effects in the guts of tumor‐burdened mice, they induce various on‐target dose‐limiting toxicities, including bone fractures.^[^ ^20^ ^]^ Using a 3D‐organoid model, some researchers have demonstrated that 70% of CRC cases bearing APC mutations exhibit a favorable growth status independent of the Wnt pathway activators,^[^ ^20^, ^21^ ^]^ which further limits the efficacy of Wnt pathway inhibitors. Despite the potential of the pharmacological inhibition of Wnt signaling as a therapeutic option for patients with cancer, finding effective and low‐toxicity inhibitors of this pathway remains a significant challenge. Notably, some inhibitors targeting the Wnt pathway, such as adavivint and teplinovivint, have advanced to phase II clinical trials.^[^ ^22^ ^]^ These compounds suppress the Wnt signaling pathway through other bypasses or upstream regulatory pathways, thereby exerting fewer side effects.

Recently, 3D‐organoid culture technology for tumors has been developed that closely recapitulates the properties of the original tumor and provides significant advantages for biological and drug research on cancer.^[^ ^23^ ^]^ Han et al. revealed that CRC organoids bearing mutations in the transforming growth factor‐beta, TP53, and Hippo pathways exhibit resistance to Wnt pathway inhibitors,^[^ ^20^ ^]^ suggesting the need for pathway‐targeted treatment. In this study, through transcriptomic analysis of CRC organoids and tissues, we found that the transcriptional activation of Wnt pathway in CRC was independent of the canonical Wnt/β‐catenin pathway and associated with liver metastasis. Subsequently, high‐throughput screening of inhibitors targeting the Wnt pathway using an organoid model revealed that adavivint not only suppressed CRC organoid growth but also showed low toxicity to colorectal organoids. Mechanistically, adavivint blocked the transcription of Wnt target genes by bypassing ADAM10/NOTCH2 signaling, resulting in growth arrest and susceptibility to chemotherapy. This study provides a promising small‐molecule inhibitor and therapeutic target for CRC.

## 2 Results

### 2.1 Transcriptional Regulation of the Wnt Pathway, Independent of the Canonical Wnt/β‐Catenin Pathway, is Activated and Associated with Liver Metastasis in CRC

**Figure 1:**
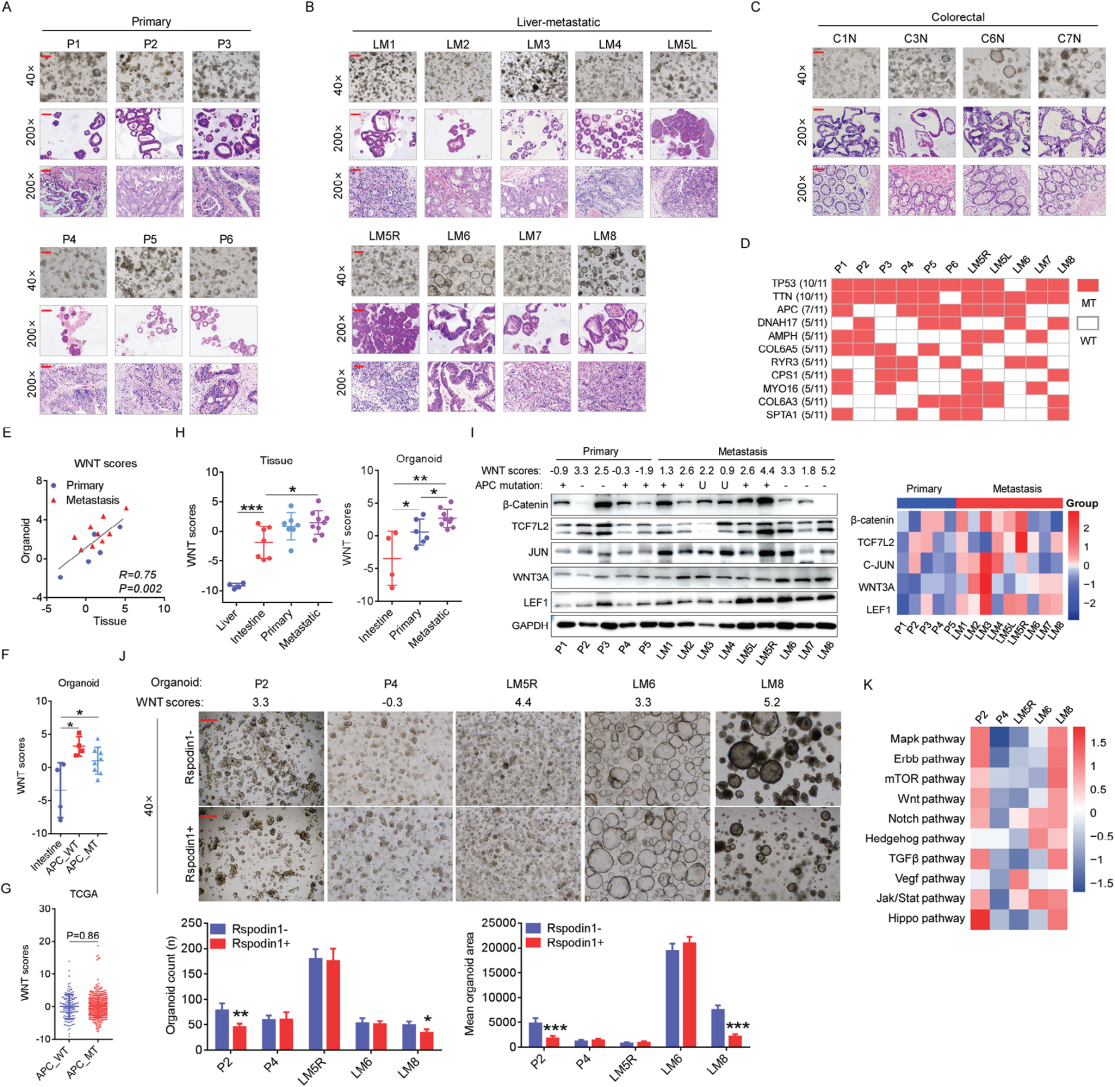
Transcriptional regulation of Wnt pathway is activated and associated with liver metastasis in CRCs. A–C) Establishment of primary CRC‐derived, liver‐metastatic CRC‐derived and colorectal organoids. Representative bright‐field microscopy images of six organoids derived from primary CRC tissues (P1‐P6), nine organoids derived from liver‐metastatic CRC tissues (LM1‐LM8) and four colorectal organoids derived from colorectal tissues (C1N,C3N,C6N,C7N), and HE staining of these organoids and corresponding tissues. D) Top 10 mutated genes in established organoids by WES sequencing. E) Correlation analysis reveals positive relationships of Wnt score between organoids and paired tumor tissues (n = 14). F) Expression of enrichment scores of Wnt pathway in colorectal organoids (n = 4), APC wild‐type CRC organoids (n = 4) and APC‐mutant CRC organoids (n = 8). G) Expression of enrichment score of Wnt pathway in APC wild‐type (n = 142) and APC‐mutant CRC tissue (n = 391) in TCGA database. H) Expression of enrichment scores of Wnt pathway in liver‐metastatic CRC tissues (n = 9), liver tissues (n = 4), normal colorectal tissues (n = 7) and primary CRC tissues (n = 7), or in colorectal organoids (n = 4), primary CRC organoids (n = 6) and liver‐metastatic CRC organoids (n = 9). I) Western blot analysis of members of classical Wnt pathway in 5 primary CRC organoids and 9 liver‐metastatic CRC organoids (Left). WNT scores and APC mutation are also displayed. Symbol “U” indicates the mutation is unknown. Heatmap indicates the relative protein levels of β‐catenin, TCF7L2, JUN, WNT3A and LEF1 (Right). J) Bright field photograph of the indicated CRC organoids cultured in medium with or without R‐spondin1. K) Heatmap shows changed enrichment scores of the indicated pathways after treated by medium minus R‐spondin1. Changed enrichment score = enrichment scores of pathways in organoids cultured medium minus R‐spondin1 – enrichment scores of pathways in organoids cultured medium plus R‐spondin1. “MT” or “WT” represents “mutation” or “wild type” respectively. WNT scores indicate Wnt pathway enrichment scores by GSVA analysis. Bar: 40×, 200µm; 200×, 50µm. All P‐values in (F, G, and H) are calculated using an unpaired two‐sided Student's t‐test, and data presented as mean ± SD. *p < 0.05, **p < 0.01, ***p < 0.001.

Organoid culture could obtain high‐purity cancer cells compared with organoid‐derived tumor tissue and favor studies focusing on cancer cells by excluding disruption of the microenvironment. We successfully generated six primary CRC organoids from six primary CRC samples (**Figure** **1**A), nine liver metastatic CRC organoids from eight liver metastatic CRC samples (Figure 1B), and four colorectal organoids from four normal colorectal tissues (Figure 1C). Hematoxylin and eosin (HE) staining of the organoids and corresponding tissues was performed for pathological identification. Transcriptomic (Figure S1A, Supporting Information) and whole exome sequencing analyses (Figure S1B, Supporting Information) were performed to demonstrate that the organoids preserved the transcriptomic and genomic characteristics of the derived tumor tissues. Over 90% of the CRC organoids showed either TP53 or APC mutations (Figure 1D). Through gene set variation analysis (GSVA) using RNA‐seq data, enrichment score of Wnt pathway (name as “WNT score”) could be calculated. By performing a correlation analysis of WNT scores between CRC tissues and CRC organoids, we found that CRC organoids could mimic the transcriptional activity of the Wnt pathway in CRC tissues (Figure 1E). We observed higher transcriptional activity of the Wnt pathway in CRC organoids than in colorectal organoids; however, transcriptional regulation of the Wnt pathway was not specifically activated in APC‐mutant CRC organoids (Figure 1F). On the Cancer Genome Atlas (TCGA) database, enrichment scores of the Wnt pathway in APC wild‐type and APC‐mutant CRC tissues were similar (Figure 1G). Further analysis revealed a higher transcriptional activity of the Wnt pathway in liver metastatic CRC than in colorectum or primary CRC (Figure 1H). The protein levels of Wnt pathway members in five primary CRC organoids and nine liver metastatic CRC organoids were detected by western blotting. Further quantitative analysis revealed elevated levels of WNT3A, LEF1, and JUN in liver metastatic CRC organoids (Figure 1I). We noted that the P2 and LM8 organoids with high WNT scores expressed little β‐catenin (Figure 1I). We then used the medium without the Wnt pathway activator RSPO1 to culture CRC organoids and found that these organoids grew well in the medium minus RSPO1 (Figure 1J). Interestingly, the P2 and LM8 organoids grew faster in the medium without RSPO1 than in the medium containing RSPO1. RNA‐seq and Kyoto Encyclopedia of Genes and Genomes (KEGG) pathway analyses revealed that the P2 and LM8 organoids showed enhanced transcriptional regulation of the Wnt and other tumor‐associated pathways, especially the Notch and Jak/Stat pathways, in the medium without RSPO1 (Figure 1K). These results suggest that transcriptional regulation of Wnt pathway is activated in CRC, especially in liver‐metastatic CRC, and regulated independent of the canonical Wnt/β‐catenin pathway.

### 2.2 High‐Throughput Screening Using CRC and Colorectal Organoid Models Revealed Adavivint as a Therapeutic Small‐Molecule Inhibitor Targeting the Wnt Pathway

**Figure 2:**
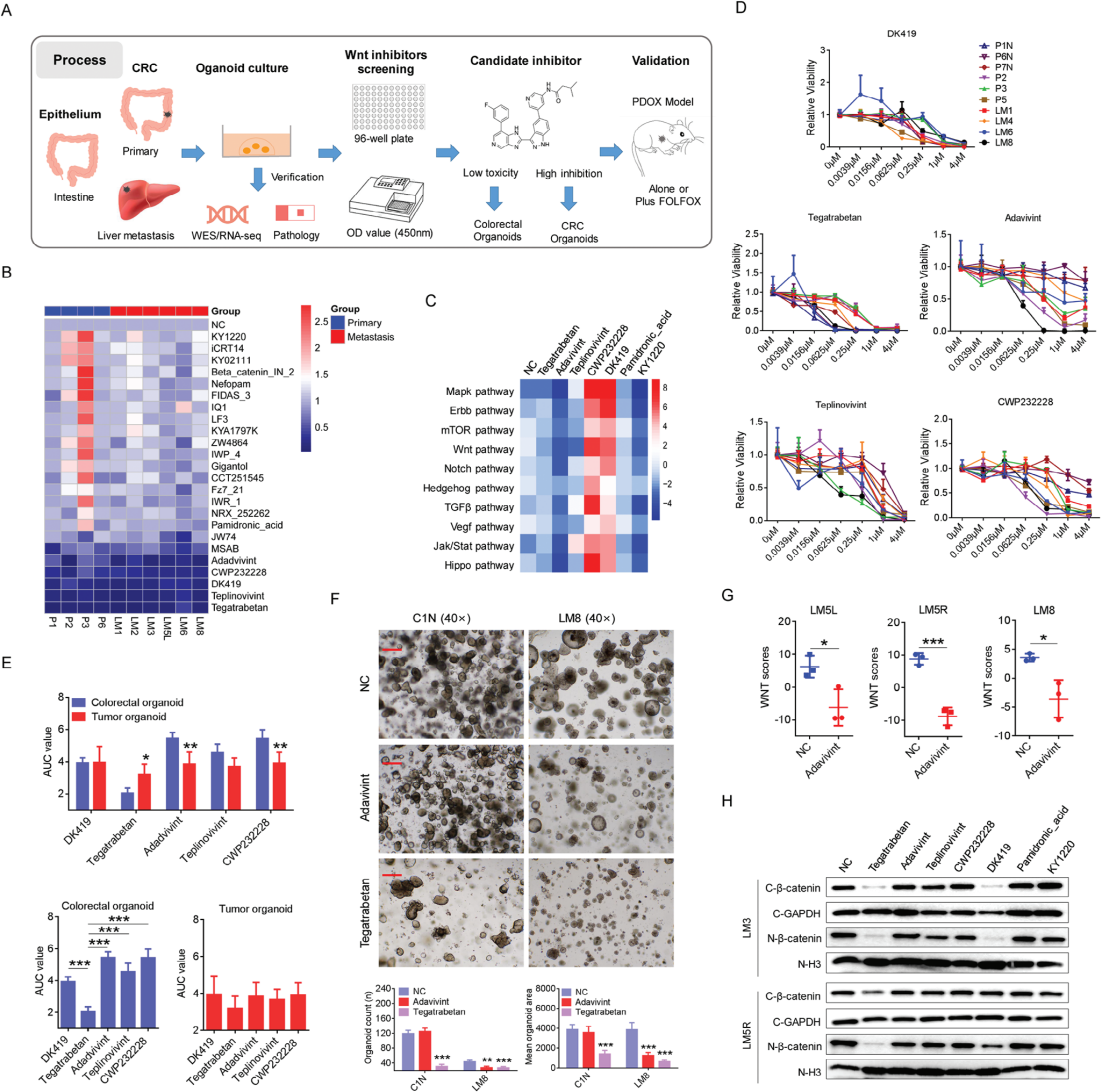
Usage of CRC organoid model to screen inhibitors targeting transcriptional regulation of Wnt pathway. A) Process using CRC and colorectal organoids to screening inhibitors targeting Wnt pathway. B) Heatmap shows relative viability of the 24 indicated inhibitors targeting Wnt pathway at the concentration of 1 µM. C) Heatmap shows the changed enrichment scores of KEGG pathway in LM3 organoids treated by the indicated 7 inhibitors (1 µM) for 24 hours. Changed enrichment score = enrichment scores of pathways in inhibitors‐treated organoids – enrichment scores of pathways in control organoids. D) Dose‐response curves for the 5 indicated compounds in 3 colorectal organoids, 3 primary CRC organoids and 4 liver‐metastatic CRC organoids. E) Histogram shows AUC values of the 5 indicated compounds in CRC organoids (n = 7) and colorectal organoids (n = 3). F) Representative bright‐field microscopy images of colorectal organoids and CRC organoids treated by adavivint and tegatrabetan (1 µM) for 96 hours (n = 4). Bar: 40×, 200µm.G) Enrichment scores of Wnt pathway in LM5L, LM5R and LM8 organoids treated by adavivint (0.5 µM) for 24 hours (n = 3). H) Western blot shows the protein expression of β‐catenin in cytoplasm and nucleus after treated by tegatrabetan, adavivint, teplinovivint, CWP232228, DK419, pamidronic_acid and KY1220 for 24 hours. All P‐values in (E, F and G) are calculated using an unpaired two‐sided Student's t‐test, and data presented as mean ± SD. *p < 0.05, **p < 0.01, ***p < 0.001.

Given the proven specificity of the activated transcription of the Wnt pathway in CRC, we searched for 24 inhibitors targeting this pathway (Table S1, Supporting Information). Among these compounds, we found that some suppressed the Wnt pathway were nor clearly clarified, such as adavivint and teplinovivint. These compounds may suppress the Wnt pathway at the transcriptional level through other pathways. **Figure** **2**A provides detailed information on our screening process using established organoids, and the heatmap in Figure 2B illustrates the relative viability of the 24 inhibitors after a 96‐h treatment at a concentration of 1 µM. Of the 24 small‐molecule inhibitors, tegatrabetan, adavivint, teplinovivint, CWP232228 and DK419 exhibited the highest inhibitory activity against CRC organoids. JW74 and MSAB showed slight inhibitory effects on CRC organoids, and other 19 inhibitors did not showed toxicity on CRC organoids, including pamidronic acid, and KY1220. To explore the action mechanisms of these compounds, we conducted RNA‐seq analysis on CRC organoids treated with tegatrabetan, adavivint, teplinovivint, CWP232228, DK419 at 1 µM for 24 h. Two inhibitors, pamidronic acid, and KY1220 who did not inhibit the growth of CRC organoids, were also included. GSVA of the KEGG pathway revealed that tegatrabetan, adavivint and teplinovivint suppressed transcriptional regulation of the Wnt pathway. Among the pathways known to be involved in tumor development, tegatrabetan and adavivint effectively inhibited the Hedgehog and Notch pathways (Figure 2C). However, the administration of CWP232228 and DK419 reversed the changes in the transcription levels of these pathways. For example, CWP232228, specifically antagonizing binding of β‐catenin to T‐cell factor (TCF) in the nucleus, activated transcriptional activation of Wnt pathway and Notch pathway. Subsequently, we conducted a more detailed examination of the inhibition ratios of various concentrations of tegatrabetan, adavivint, teplinovivint, CWP232228, and DK419 in colorectal, primary CRC, and metastatic CRC organoids (Figure 2D). Area under the curve (AUC) values revealed that adavivint not only inhibited CRC organoid growth but also showed low toxicity to colorectal organoids (Figure 2E). Tegatrabetan, specifically disrupting the binding of β‐catenin with the scaffold protein transducin β‐like 1, also suppressed CRC organoids growth among the five compounds, but caused high toxicity on normal colorectal organoids (Figure 2E,F). RNA‐seq and GSVA revealed that adavivint suppressed the Wnt pathway at the transcriptional level in CRC organoids (Figure 2G). However, nucleus entrance of β‐catenin was not notably attenuated after 24‐h treatment with adavivint (Figure 2H).

### 2.3 CRC with High Transcriptional Activity of the Wnt Pathway is Vulnerable to Adavivint In Vitro and In Vivo

**Figure 3:**
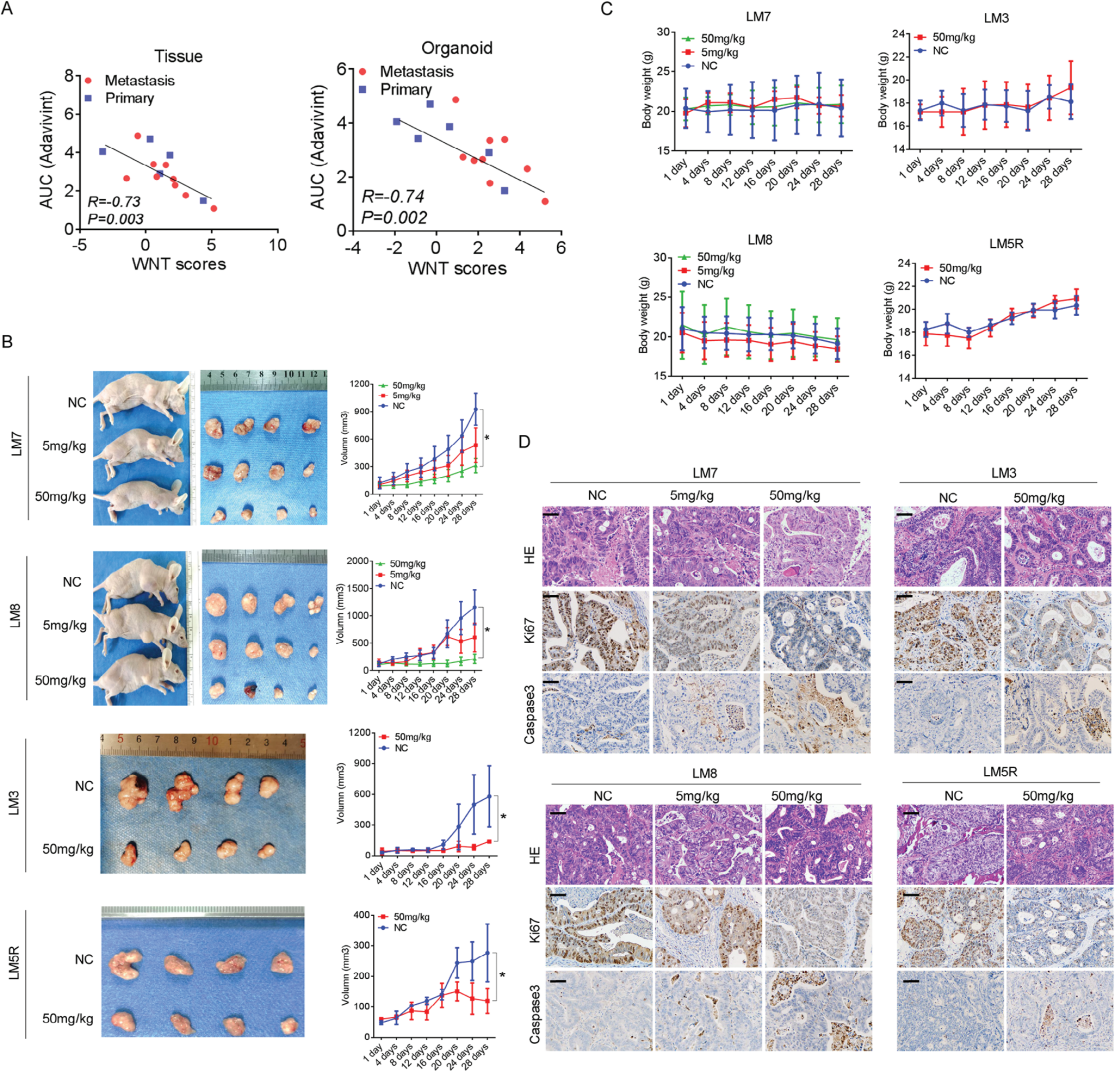
Adavivint suppresses CRCs growth in vitro and in vivo. A) Correlation analysis between enrichment scores of Wnt pathway and AUC value of adavivint in CRC tissues (R = ‐0.73. P = 0.003, n = 14) and organoids (R = ‐0.74. P = 0.002, n = 15). Spearman correlation. The effect of adavivint on tumor growth B, n = 4) or body weight C, n = 4) of PDOX model established using the indicated organoids at the dose of 5mg kg^−1^ or 50mg kg^−1^. Data presented as mean ± SD, all P‐values are calculated using an unpaired two‐sided Student's t‐test. *p < 0.05. D) HE staining and IHC analysis for Ki67 and Caspase3 in representative sections from the PDOX model established using the indicated organoids. Bar: 200×, 50µm.

Next, dose–response curves were plotted for adavivint in CRC organoids (Figure S2A, Supporting Information). Correlation analysis showed a negative relationship between the enrichment score of the Wnt pathway and the AUC of adavivint in tumor tissues and organoids (**Figure** **3**A). We also established two lung adenocarcinoma (LUAD) and pancreatic adenocarcinoma (PDAC) organoids (Figure S2B,C, Supporting Information), and the organoids with high transcriptional levels of the Wnt pathway were sensitive to adavivint (Figure S2D,E, Supporting Information). To explore the inhibitory effect on CRC growth in vivo, a patient‐derived organoid xenograft (PDOX) model was constructed using CRC organoids with high enrichment scores for the Wnt pathway, and two doses of adavivint (5 and 50 mg kg^−1^) were administered. Adavivint effectively suppressed CRC growth (Figure 3B) but did not affect the body weight of tumor‐bearing mice (Figure 3C) at a dose of 50 mg kg^−1^; similar responses were observed in the two other CRC organoids in vivo (Figure 3B,C). HE staining and immunohistochemistry (IHC) analysis revealed low levels of Ki67 and high levels of caspase 3 in the adavivint‐treated PDOX model (Figure 3D).

### 2.4 ADAM10/NOTCH2 Signaling is a Target of Adavivint to Regulate the Wnt Pathway Transcription in CRC

**Figure 4:**
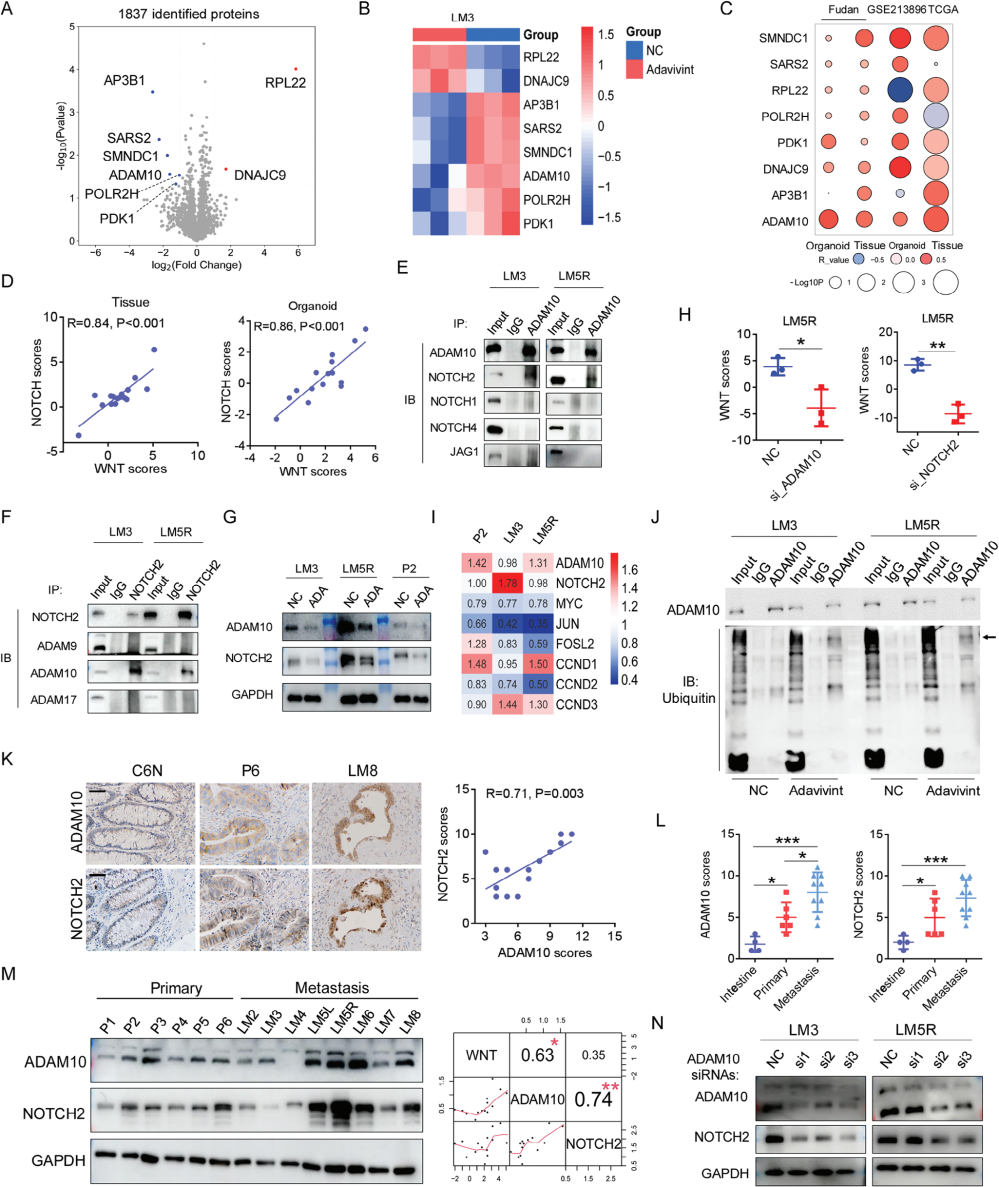
ADAM10/NOTCH2 signaling is identified as a target of adavivint to regulate Wnt pathway in CRCs. A) Volcano plot B) Heatmap shows the 6 significantly changed proteins (1.5 or 0.67, P < 0.05) after adavivint treatment for 24 hours in P9 organoids. C) Bubble diagram shows relationships between RNA levels of the six genes and Wnt pathway enrichment scores in CRCs in the indicated datasets. D) Correlation analysis of enrichment scores of Wnt pathway and NOTCH pathway in CRC tissues (R = 0.84. P < 0.001, n = 14) and organoids (R = 0.86. P < 0.001, n = 15). Spearman correlation. E) NOTCH receptors and NOTCH ligands are detected by Western blot after immunoprecipitated by anti‐ADAM10 body. F) Members of ADAM family are detected by Western blot after immunoprecipitation assay using anti‐NOTCH2 body. G) ADAM10 and NOTCH2 are detected by Western blot after treatment of adavivint for 24 hours. “ADA” indicates “adavivint”. H) Wnt pathway enrichment analysis after knockdown of ADAM10 or NOTCH2 in the indicated organoids (n = 3). I) Heatmap shows relative fold change of ADAM10, NOTCH2 and Wnt target genes by qRT‐PCR after treated by adavivint for 24 hours. J) Cell lysates are analyzed by immunoprecipitation with anti‐ADAM10 and Western blot with indicated antibodies. The black arrow indicates the band location of ADAM10 protein. K) IHC analysis for ADAM10 and NOTCH2 in representative sections from the normal tissue, primary CRC tissue, and liver‐metastatic CRC tissue (left), and correlation analysis (Spearman correlation, R = 0.86. P<0.001, n = 15) using their scores by IHC analysis in CRC tissue (right). Bar: 200×, 50µm. L) Histogram shows relative protein levels of ADAM10 and NOTCH2 in normal colorectal (n = 4), primary (n = 6) and metastatic tumor tissues (n = 6) by IHC analysis. M) ADAM10 and NOTCH2 are detected by Western blot in the indicated organoids (Left), and Corrplot shows the relationships among Wnt pathway enrichment scores, relative protein levels of ADAM10 and NOTCH2 in CRC organoids (Right). N) After knockdown of ADAM10, ADAM10 and NOTCH2 are detected by Western blot in the indicated organoids. All P‐values in (H and L) are calculated using an unpaired two‐sided Student's t‐test, and data presented as mean ± SD. *p < 0.05, **p < 0.01, ***p < 0.001.

To comprehensively elucidate the mechanism of adavivint‐mediated growth inhibition, we conducted mass spectrometric analysis at 24‐h post‐treatment with adavivint. Although 1837 proteins were identified (**Figure** **4**A), only six significantly changed proteins were found and are displayed in the heatmap (fold change >1.5 or <0.67, P< 0.05; Figure 4B). CLK2 and DYRK1A, the targets of adavivint reported in previous study,^[^ ^22^, ^24^ ^]^ were not detected by mass spectrometric analysis in this study. Subsequently, correlation analysis was performed to unveil the positive relationship between ADAM10 and the Wnt pathway using RNA‐seq data from organoids and tissues, along with two additional RNA‐seq datasets from TCGA and GEO databases (Figure 4C). To further investigate the mechanism by which ADAM10 regulates the Wnt pathway, we calculated the enrichment scores of 189 KEGG pathways and conducted a correlation analysis between these pathways and ADAM10 mRNA levels in CRC organoids (Table S2, Supporting Information). Subsequently, two pathways were identified, with only the NOTCH pathway showing a significant increase in transcriptional levels in liver metastatic organoids and tissues (Figure S3A, Supporting Information). Correlation analysis revealed positive relationships between the Wnt and Notch pathways in CRC tissues and organoids (Figure 4D). In the IP assay, we found that only ADAM10 directly bound to NOTCH2 but not to other members of the NOTCH receptors and ligands (Figure 4E). Other ligands of NOTCH receptors, such as delta‐like (DLL)‐1, DLL3, DLL4 and Jagged2 (JAG2) could hardly were not detected by western blotting in CRC organoids (data not shown). Among the three members of the ADAM family, only ADAM10 was immunoprecipitated using the NOTCH2 antibody (Figure 4F). Furthermore, western blotting analysis revealed significant downregulation of ADAM10 and NOTCH2 levels (Figure 4G) but no significant changes in NOTCH1, NOTCH4, ADAM9, and ADAM17 levels after adavivint treatment for 24 h (Figure S3B, Supporting Information). Organoids treated with siRNAs targeting ADAM10 and NOTCH2 for 72 h showed a significant decrease in the transcriptional levels of the Wnt pathway, as determined by RNA‐seq analysis (Figure 4H). Moreover, downstream genes of the Wnt pathway were significantly decreased after adavivint treatment for 24 h, including MYC, JUN, and CCND2, whereas the mRNA levels of ADAM10 and NOTCH2 did not exhibit a significant decrease (Figure 4I). Immunoprecipitation (IP) assay with the anti‐NOTCH2 antibody revealed that adavivint enhanced the ubiquitination of ADAM10 (Figure 4J). These results suggest that adavivint suppresses the regulation of the Wnt pathway by specifically attenuating the protein expression of ADAM10 and NOTCH2.

Next, IHC was performed to analyze the expression levels of ADAM10 and NOTCH2 in the normal colorectal and primary and metastatic CRC tissues (Figure 4K). IHC revealed a positive association between ADAM10 and NOTCH2 expression levels (Figure 4K), with the highest levels of ADAM10 and NOTCH2 proteins detected in the liver metastatic CRC tissues compared to those in the normal colorectal and primary CRC tissues (Figure 4L). We also assessed the protein levels of ADAM10 and NOTCH2 in CRC organoids via western blotting analysis (Figure 4M). Correlation analysis revealed positive relationships between ADAM10, NOTCH2, and Wnt pathway enrichment scores (Figure 4M). *ADAM10* knockdown decreased the protein levels of NOTCH2 in CRC organoids (Figure 4N).

### 2.5 ADAM10/NOTCH2 Signaling Functions as a Bypass Signaling to Regulate Wnt Pathway by Inducing the Transcription of TCF7L2 and Wnt Target Genes in CRC

**Figure 5:**
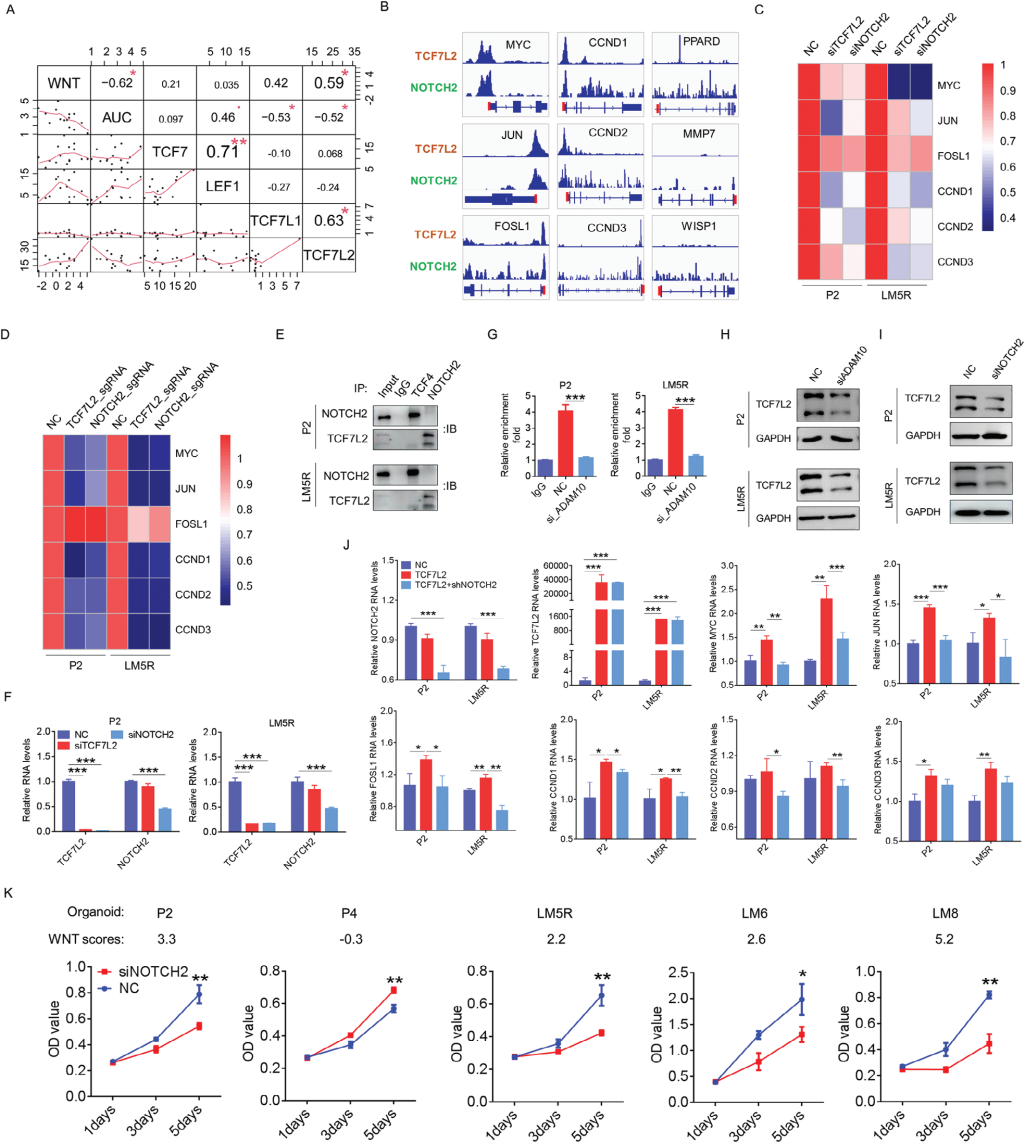
ADAM10/NOTCH2 signaling directly activates transcription of TCF7L2 and Wnt target genes in CRC. A) Corrplot shows the relationships among AUC of adavivint, Wnt pathway enrichment score and mRNA level of transcription factor of Wnt pathway downstream in CRC organoid (n = 15, spearman correlation). B) In LM5R organoids, Cut&tag assay and sequencing analysis indicated that TCF7L2 and NOTCH2 could be enriched in promoters of Wnt target genes. The red bar indicates gene start site. C) Relative fold change of mRNA levels of the Wnt target genes after NOTCH2 or TCF7L2 knockdown using siRNAs in the indicated CRC organoids. D) Relative fold change of mRNA levels of the Wnt target genes after NOTCH2 or TCF7L2 stable knockdown in the indicated CRC organoids. E) Co‐IP assay of NOTCH2 and TCF7L2 in the indicated organoids. F) Relative fold change of mRNA levels of NOTCH2 or TCF7L2 after NOTCH2 or TCF7L2 knockdown in the indicated CRC organoids (n = 3). G) Cut&tag assay and qRT‐PCR shows that NOTCH2 directly binds to promoter of TCF7L2, which could be attenuated by ADAM10 knockdown (n = 3). H,I) Western blot shows decreased TCF7L2 after ADAM10 and NOTCH2 knockdown in the indicated CRC organoids. J) Relative mRNA levels of the Wnt target genes after NOTCH2 knockdown in CRC organoids overexpressed TCF7L2 (n = 3). K) CCK8 assay is conducted to assess proliferation ability of the indicated organoids treated by NOTCH2 siRNAs (n = 3). All P‐values in (F, G, J, and K) are calculated using an unpaired two‐sided Student's t‐test, and data presented as mean ± SD. *p < 0.05, **p < 0.01, ***p < 0.001.

To elucidate the potential mechanisms through which NOTCH2 regulates the classical Wnt pathway, we initially conducted a correlation analysis among the enrichment score of the Wnt pathway, AUC of adavivint, and the mRNAs of the four transcription factors of the classical Wnt pathway (TCF7, TCF7L2, TCF7L1, and LEF1). TCF7L2 significantly correlated with the transcriptional level of the Wnt pathway and adavivint sensitivity (**Figure** **5**A). Of the four transcription factors, TCF7L2 exhibited the highest mRNA levels in CRC tissues and organoids (Figure S4, Supporting Information). We also conducted Cut&Tag assays targeting NOTCH2 and TCF7L2. Of the nine downstream genes of the classical Wnt pathway, the results indicated peak enrichment of both NOTCH2 and TCF7L2 at the MYC, JUN, FOSL1, CCND1, CCND2, and CCND3 promoters (Figure 5B).

To clarify the regulatory mechanisms by which TCF7L2 and NOTCH2 control downstream genes in the classical Wnt pathway, we initially knocked down TCF7L2 and NOTCH2 in CRC organoids (Figure S5A,B, Supporting Information), which decreased the expression levels of MYC, JUN, FOSL1, CCND1, CCND2, and CCND3 (Figure 5C). Similar results were observed in stable knockdown CRC organoids using lentiviruses containing sgRNAs targeting TCF7L2 and NOTCH2 (Figure S5C, D, Supporting Information; Figure 5D). However, co‐IP assay using NOTCH2 and TCF7L2 antibodies revealed no direct interaction between NOTCH2 and TCF7L2 (Figure 5E). A previous study also reported that the NOTCH2 ligand, DLL1, regulates gene transcription by interacting with other transcriptional factors, such as TCF7L2 and Smad2/3^[^ ^25^ ^]^. However, IP assay of the TCF7L2 antibody indicated no direct interplay between TCF7L2 and other NOTCHs or their ligands in this study (due to the extremely low levels, other ligands, such as JAG2, DLL1, and DLL3, were not detected; Figure S6, Supporting Information). *NOTCH2* knockdown decreased the TCF7L2 mRNA levels in CRC organoids (Figure 5F). Based on the results of the cut‐and‐tag and sequencing analyses, a pair of specific primers was designed. Cut&Tag and quantitative reverse transcription‐polymerase chain reaction (qRT‐PCR) assays revealed the direct interaction between NOTCH2 protein and TCF7L2 promoter, which was attenuated by *ADAM10* knockdown (Figure 5G). TCF7L2 protein levels were also decreased by *ADAM10* and *NOTCH2* knockdown (Figure 5H–I). Furthermore, TCF7L2‐mediated expression of MYC, JUN, FOSL1, CCND1, and CCND2 was reversed by *NOTCH2* knockdown in CRC organoids (Figure 5J). NOTCH2 siRNA treatment of CRC organoids with different WNT scores revealed the growth rate of organoids with high transcriptional levels of the Wnt pathway, such as P1, LM6, LM5R, and LM8 (Figure 5K). However, P4 organoids with lower enrichment scores for the Wnt pathway grew faster after treatment with NOTCH2 siRNA (Figure 5K). These results suggest ADAM10/NOTCH2/TCF7L2 as a bypass signaling pathway that regulates the transcription of Wnt target genes.

Cut&Tag assays also revealed enriched peaks in the promoters of ADAM10 and JAG1 (Figure S7A, Supporting Information). qRT‐PCR (Figure S7B, Supporting Information) and western blotting (Figure S7C, Supporting Information) revealed that both *TCF7L2* and *NOTCH2* knockdown decreased the expression of JAG1, but not ADAM10. Adavivint also decreased JAG1 expression in CRC organoids (Figure S7D, Supporting Information).

### 2.6 Use of Adavivint and Blockage of ADAM10/NOTCH2/TCF7L2 Signaling Suppress CRC Growth by Inhibiting Wnt Target Gene Expression

**Figure 6:**
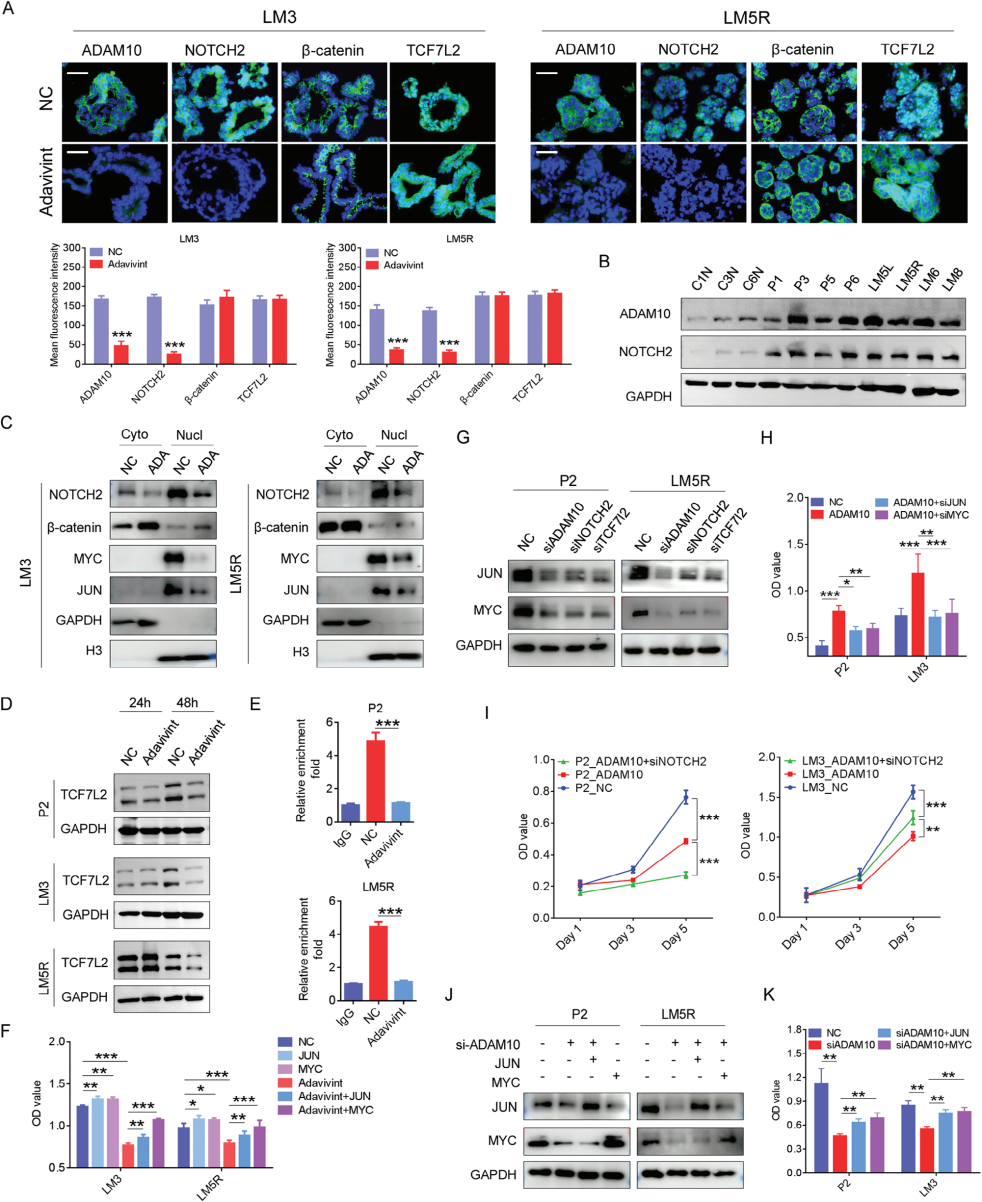
Both adavivint and blockage of ADAM10/NOTCH2/TCF7L2 signaling suppress growth of CRC organoids by decreasing expression of Wnt target genes. A) IF analysis for ADAM10, NOTCH2, β‐catenin and TCF7L2 in CRC organoids after adavivint treatment for 24 hours (n = 4). Bar: 400×, 50µm. B) Western blot analysis of ADAM10 and NOTCH2 in colorectal tissues, primary and liver‐metastatic CRC tissues. C) Western blot analysis of NOTCH2, β‐catenin, MYC and JUN in the indicated organoids after treated by adavivint (0.5 µM) for 24 hours. “ADA” indicates “adavivint”. D) Western blot analysis of TCF7L2 in the indicated organoids treated by adavivint (0.5 µM) for 24 hours and 48 hours. E) Cut&tag assay and qRT‐PCR shows that NOTCH2 binding to promoter of TCF7L2 could be attenuated by adavivint (0.5 µM) for 24 hours (n = 3). F) After treated by adavivint for 24 hours, CCK8 assay is performed to examine the proliferation ability of the indicated organoids transfected by MYC or JUN‐overexpressing plasmid (n = 4). G) Western blot analysis of MYC and JUN in the indicated organoids transfected by ADAM10, NOTCH2, and TCF7L2 siRNAs. H) In the indicated organoids transfected by ADAM10‐overexpressing plasmid for 24 hours, CCK8 assay is performed to assess the proliferation ability of the indicated organoids after transfected by MYC or JUN siRNA. I) CCK8 assay was used to examine the effect of NOTCH2 knockdown on proliferation ability of the indicated organoids overexpressing ADAM10 (n = 4). In the indicated organoids transfected by ADAM10 siRNA for 24 hours, J) Western blot analysis was used to detect MYC and JUN after transfected by MYC or JUN‐overexpressing plasmid, and K) CCK8 assay is further performed to assess the proliferation ability of the indicated organoids (n = 3). All P‐values in are calculated using an unpaired two‐sided Student's t‐test, and data presented as mean ± SD. *p < 0.05, **p < 0.01, ***p < 0.001.

IF analysis revealed decreased expression of ADAM10 in the cytomembrane and cytoplasm, as well as decreased expression of NOTCH2 in the cytomembrane, cytoplasm, and nucleus after 24‐h of adavivint treatment (**Figure** **6**A). However, the expression of β‐catenin and TCF7L2 remained unchanged (Figure 6A). Compared to primary and liver metastatic CRC tissues, normal colorectal tissues showed lower levels of ADAM10 and NOTCH2 (Figure 6B). By nuclear cytoplasmic separation assay, we also observed decreased NOTCH2 in both the cytoplasm and nucleus, with no obvious change in the nuclear entrance of β‐catenin, as shown by western blotting (Figure 6C). Moreover, we did not detect MYC and JUN expression in the cytoplasm, but observed decreased expression in the nucleus (Figure 6C). Adavivint did not change TCF7L2 expression after 24 h of treatment, but decreased TCF7L2 expression at 48 h (Figure 6D). Using the Cut&Tag and qRT‐PCR assays, NOTCH2 binding to the TCF7L2 promoter was attenuated by adavivint treatment for 24 h (Figure 6E). Forcing MYC and JUN expression rescued the adavivint‐induced growth suppression of CRC organoids (Figure 6F).

Next, we examined the decreased protein levels of MYC and JUN in CRC organoids treated with ADAM10, NOTCH2, and TCF7L2 siRNAs (Figure 6G). To explore whether NOTCH2, MYC, and JUN mediate ADAM10‐induced organoid growth, we upregulated the expression of ADAM10 in CRC organoids (Figure S5E, Supporting Information) and observed the outgrowth of CRC organoids (Figure 6H). This outgrowth was reversed by *MYC*, *JUN*, and *NOTCH2* knockdown (Figure 6H,I; Figure S5F, G, Supporting Information). *ADAM10* knockdown attenuated the effects of MYC and JUN overexpression (Figure 6J,K).

### 2.7 Use of Adavivint and Blockage of ADAM10/NOTCH2 Pathway Alleviate CRC Chemoresistance by Inhibiting Wnt Target Gene Expression

**Figure 7:**
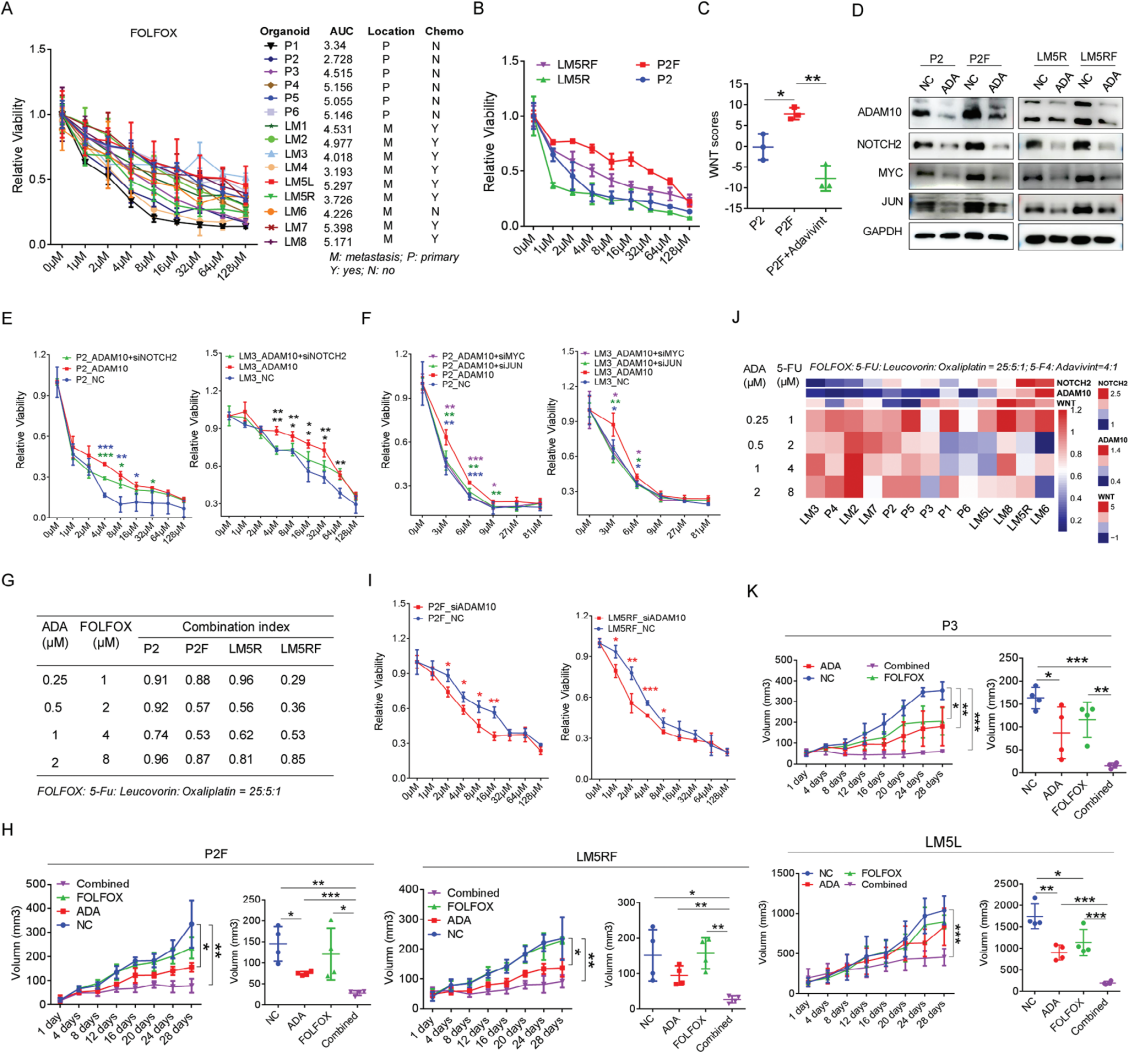
Adavivint or targeting ADAM10/NOTCH2 signaling alleviates chemoresistance by suppressing expression of Wnt target genes in CRCs. A) CCK8 assay was performed to calculate the AUC of FOLFOX in the indicated organoids. “Chemo” with “Y” indicated the patient had received regimen of FOLFOX before surgery. B) Dose‐response curves for regimen of FOLFOX in established FOLFOX‐resistant CRC organoids (n = 3). C) Enrichment scores of Wnt pathway in FOLFOX‐resistant CRC organoids treated by adavivint (0.5 µM, n = 3). D) In FOLFOX‐resistant organoids, Western blot analysis is used to detect ADAM10, NOTCH2, MYC and JUN after treated by adavivint for 24 hours. “ADA” indicates “adavivint”. E) CCK8 assay is conducted to examine the survival inhibition ratio of FOLFOX regimen in the indicated organoids transfected by ADAM10‐overexpressing plasmid and NOTCH2 siRNA (n = 3). F) After transfected by ADAM10‐overexpressing plasmid, CCK8 assay is used to examine the survival inhibition ratio of regimen in the indicated organoids treated by MYC siRNA or JUN siRNA (n = 3). G) Synergistic index of adavivint and FOLFOX is calculated in the indicated dose in FOLFOX‐resistant organoids. H) The effect of combination of adavivint and FOLFOX on the growth of tumor in PDOX model established using the FOLFOX‐resistant organoids (n = 4). I) CCK8 assay is examine the relative viability of the FOLFOX‐resistant organoids transfected by ADAM10 siRNA (n = 3). J) Heatmap shows the synergistic index of adavivint and FOLFOX at different concentration in the indicated organoids. “ADA” indicates “adavivint” K) The effect of combination of adavivint and FOLFOX on the growth of tumor in PDOX model established using the indicated organoids (n = 4). All P‐values in are calculated using an unpaired two‐sided Student's t‐test, and data presented as mean ± SD. *p < 0.05, **p < 0.01, ***p < 0.001.

We demonstrated that adavivint inhibited CRC organoid growth by interfering with the Wnt pathway. Activation of the Wnt pathway is associated with chemoresistance in CRC.^[^ ^26^, ^27^ ^]^ Therefore, we investigated whether adavivint alleviates chemoresistance in CRC. Initially, we assessed the sensitivity of CRC organoids to the folinic acid, fluorouracil, and oxaliplatin (FOLFOX) regimen, which revealed diverse responses among different organoids (**Figure** **7**A). Then, we successfully generated FOLFOX‐resistant organoids using the P2 and LM5R organoids, named P2F and LM5RF, respectively (Figure 7B). After the induction of FOLFOX resistance, enrichment score of the Wnt pathway increased but was reversed by adavivint in P2 organoids (Figure 7C). These two FOLFOX‐resistant organoids also exhibited elevated expression levels of ADAM10, NOTCH2, MYC, and JUN, but this trend reversed by adavivint treatment (Figure 7D). In the P2 and LM3 organoids, ADAM10 overexpression induced resistance to the FOLFOX regimen, which was mitigated by the knockdown of *NOTCH2* (Figure 7E), *MYC*, and *JUN* (Figure 7F). For *MYC* and *JUN* knockdown, we treated the CRC organoids with three siRNAs and selected the most effective siRNA for subsequent experiments (Figure S5F,G, Supporting Information). By combining adavivint with the FOLFOX regimen, we observed a smaller synergistic index in the two FOLFOX‐resistant organoids than in the derived parental organoids (Figure 7G). In vivo, combined adavivint and FOLFOX regimen more effectively suppressed the growth of P2F and LM5RF organoids than any single treatment (Figure 7H). In the P2F and LM5RF organoids, *ADAM10* knockdown attenuated resistance to the FOLFOX regimen (Figure 7I). The synergistic index of adavivint and FOLFOX regimen was also calculated for other organoids, revealing synergistic effects in nearly all CRC organoids, particularly in those with high ADAM10 and NOTCH2 levels and enrichment scores of the Wnt pathway, such as the P and LM5L organoids (Figure 7J). Based on the AUC value, we selected one organoid (P3 organoid) moderately sensitive to the FOLFOX regimen and one organoid (LM5L organoid) resistant to the FOLFOX regimen to establish a PDOX model. The combined adavivint and FOLFOX regimen most effectively suppressed the growth of the P3 and LM5L organoids, with stronger effects on LM5L organoids (Figure 7K). IHC analysis of the tumors showed the lowest level of Ki67 and highest level of caspase 3 in the combined group and moderate levels in the single treatment groups (Figure S8, Supporting Information). Moreover, expression levels of ADAM10 and NOTCH2 were decreased in the adavivint and combined treatment groups (Figure S8, Supporting Information).

## 3 Discussion

CRC is associated with highly heterogeneous and complex signaling networks that significantly hinder its treatment.^[^ ^28^ ^]^ Hence, identification of critical and aberrant signaling networks, whose blockade leads to systemic failure of cancer cells, is crucial for successful treatment.^[^ ^29^ ^]^ Tumor tissues harbor a substantial number of non‐tumor cells, including immune cells and fibroblasts, which impede the identification of key signaling pathways in transcriptomic analyses. Use of organoid models can circumvent this issue and facilitate the acquisition of high‐purity cancer cells.^[^ ^30^ ^]^ Furthermore, organoid models can effectively mimic the in vivo growth status of tumor cells, thereby contributing to the identification of novel cancer driver genes and potential drug targets.^[^ ^31^ ^]^ Here, we successfully established 15 CRC organoids, including six primary CRC, nine liver‐metastatic CRC, and four colorectal organoids. CRC organoids can replicate the Wnt pathway activity at the transcriptional level in CRC tissues. Over 95% of established CRC organoids exhibit either *TP53* or *APC* mutations.^[^ ^21^, ^23^, ^32^ ^]^ Consistently, over 90% of the CRC organoids showed either *TP53* or *APC* mutations in this study.

Loss‐of‐function mutations in APC can enhance the β‐catenin/TCF transcriptional activity.^[^ ^14^ ^]^ Here, we observed higher transcriptional level of Wnt pathway in APC wild‐type and mutant CRC organoids than in the colorectal organoids. Interestingly, some CRC cases without *APC* mutation showed little expression of β‐catenin but high WNT scores and grew faster in the medium without RSPO1 than in the medium with RSPO1, indicating that the transcription of Wnt pathway is regulated independent of Wnt/β‐catenin signaling. Using transcriptome sequencing and proteomic profiling with reverse phase protein arrays, Menck et al. identified the Wnt pathway as a specific signature for CRC liver metastasis and demonstrated its association with inter‐ and intra‐patient heterogeneity.^[^ ^33^ ^]^ In this study, we also observed the activated transcription of the Wnt pathway in liver‐metastatic CRC. Targeting transcriptional regulation of Wnt pathway, different from targeting classical Wnt/β‐catenin/TCF, may be a promising therapeutic strategy to suppress CRC. Furthermore, when cultured in medium without RSPO1, organoids with high transcriptional levels of the Wnt pathway not only showed higher transcriptional activity of the Wnt pathway, but also activation of some pathways involved in tumor development, such as the Notch, Jak/Stat, and Hedgehog pathways.^[^ ^34^, ^35^, ^36^ ^]^ Maia Chanrion et al. reported that the Wnt pathway, Notch pathway, and TP53 mutations interact and maintain a balance to promote CRC progression.^[^ ^37^ ^]^ Wnt pathway involves complicated regulatory networks, and targeting this crucial point may be a key step in the successful disruption of this pathway.

To identify effective and low‐toxicity inhibitors targeting the Wnt pathway, we searched for known inhibitors, resulting in the acquisition of 24 inhibitors for subsequent drug screening. Normal colorectal organoids were used as negative controls. Notably, the specific targets of some compounds, such as adavivint and teplinovivint, are still unknown. Screening for these compounds may reveal pathways regulating the transcription of the Wnt pathway in CRC. Here, vitro and in vivo experiments revealed adavivint as a candidate regulating the transcription of the Wnt pathway. Surprisingly, its effects were not dependent on the classical Wnt/β‐catenin pathway, suggesting that adavivint disrupts transcriptional regulation of the Wnt pathway via an alternative mechanism. DK419, an inhibitor targeting the Wnt pathway by decreasing the protein levels of β‐catenin, also activates the transcription of the Wnt pathway. In biological processes, negative feedback limits the over‐activation of the Wnt pathway.^[^ ^38^, ^39^, ^40^ ^]^ DK419 targeted this pathway by suppressing the core members of Wnt pathway, such as β‐catenin, but the negative feedback mechanism‐mediated upregulation of other members of Wnt pathway limited its inhibitory effects. Targeting canonical Wnt/β‐catenin signaling by PORCN inhibitors or RSPO3 blockage leads to rapid and sustained tumor regression but also induces serious side effects.^[^ ^17^, ^18^, ^19^ ^]^ Here, small‐molecule inhibitor tegatrabetan targeting β‐catenin showed desirable anti‐tumor activity but high toxicity in normal colorectal organoids. Previous studies reported that Wnt pathway also plays crucial roles in development and therapeutic resistance of LUAD and PDAC.^[^ ^41^, ^42^ ^]^ In this study, adavivint suppressed the growth of LUAD and PDAC organoids with high enrichment scores for the Wnt pathway. Therefore, adavivint is a promising antitumor agent with few side effects for different cancer types.

Many pathways implicated in carcinogenesis and tumor development, including the TP53, Notch, Sonic Hedgehog, and Jak/Stat3 pathways, crosstalk with the classical Wnt pathway.^[^ ^12^, ^43^, ^44^ ^]^ Through mass spectrometry (MS) and transcriptomic analyses and sequential validation, we discovered that adavivint specifically attenuated ADAM10/NOTCH2 signaling at the protein level. Members of the ADAM family are multi‐domain transmembrane metalloproteases that cleave numerous cell surface proteins and activate downstream pathways involved in tumor development, including NOTCHs and their ligands.^[^ ^25^, ^45^ ^]^ IP with anti‐ADAM10 and western immunoblotting with an ubiquitin antibody indicated that adavivint degrades ADAM10 via the ubiquitin–proteasome system.^[^ ^46^ ^]^ Notably, we observed direct interactions between ADAM10 and NOTCH2; however, no direct interactions were detected between ADAM10 and the other NOTCH receptors. Members of the ADAM family, including ADAM9, ADAM10, and ADAM17, mediate signaling pathways in gastrointestinal cancer formation,^[^ ^47^ ^]^ Targeting ADAM10 and ADAM17 has demonstrated promising antitumor activity, as exemplified by compounds such as INCB3619 and INCB7839.^[^ ^48^ ^]^ Among the three ADAMs, a direct interaction was observed only between NOTCH2 and ADAM10 in CRC. In the Apc^Min/+^ model, the absence of ADAM17 resulted in a reduction in tumor formation in mouse intestinal cancer, highlighting the crucial role of ADAM17 in maintaining Wnt pathway activity.^[^ ^49^ ^]^ Initially, adavivint was discovered to block the Wnt pathway by suppressing CLK2 and DYRK1A, and has advanced phase II clinical trials for knee osteoarthritis treatment.^[^ ^22^, ^24^ ^]^ In this study, we identified 1837 proteins using MS analysis; CLK2 and DYRK1A were not detected. Thus, we identified a novel target for adavivint to regulate transcription in the Wnt pathway. Additionally, we revealed that TCF7L2 directly binds to the promoter of the NOTCH2 ligand JAG1 and promotes the transcription of JAG1, which is similar to the results of a previous study.^[^ ^27^ ^]^ Adavivint treatment also decreases JAG1 following adavivint treatment. These results suggest a novel mechanism by which adavivint inhibits transcriptional regulation of the Wnt pathway by attenuating the expression of ADAM10 and NOTCH2 proteins.

Crosstalk between the Wnt and Notch pathways plays a crucial role in maintaining stem cell‐like characteristics, as well as embryo and fetal development.^[^ ^50^, ^51^ ^]^ TCF7L2, a downstream transcription factor of the Wnt pathway, is significantly correlated with transcriptional activation of the Wnt pathway and sensitivity to adavivint. In colon carcinoma, the presence of a stable and constitutively active β‐catenin/TCF7L2 signaling pathway indicates the crucial role of TCF7L2 in carcinogenesis.^[^ ^52^, ^53^ ^]^ Among the four members of the TCF/LEF family, TCF7L2 exhibited the highest mRNA levels, indicating its pivotal role. Cut and tag assays using anti‐TCF7L2 and anti‐NOTCH2 antibodies revealed enriched peaks in the promoters of genes downstream of the Wnt pathway, including MYC, JUN, FOSL, CNND1, CNND2, and CNND3. Interestingly, we observed that *NOTCH2* knockdown reversed TCF7L2‐mediated transcriptional activation of MYC, JUN, FOSL, CNND1, CNND2, and CNND3. We further demonstrated that blocking ADAM10/NOTCH2 signaling could suppress CRC organoid growth, involving the attenuation of transcriptional activation of Wnt pathways, such as MYC and JUN. Furthermore, NICD2 the intracellular active domain of NOTCH2) binds directly to the TCF7L2 promoter and promotes the transcription and translation of TCF7L2. *NOTCH2* knockdown effectively suppresses the growth of CRC organoids at high transcriptional levels in the Wnt pathway. Similarly, Tian et al. revealed that targeting NOTUM, an extracellular palmitoleoyl‐protein carboxylesterase that antagonizes canonical Wnt signaling in mammals, is an effective approach for arresting tumor progression and metastasis in CRC.^[^ ^54^ ^]^ Additionally, we also revealed that TCF7L2 could directly bind to the promoter of NOTCH2 ligand JAG1 and promote transcription of JAG1, which was similar with previous study.^[^ ^27^ ^]^ Adavivint treatment decreased JAG1 expression in CRC organoids. Therefore, ADAM10/NOTCH2/TCF7L2 signaling serves as a bypass pathway to activate the transcription of Wnt target genes, and the Wnt pathway can be effectively disrupted by blocking this signaling pathway.

In CRC, activation of the Wnt and Notch pathways is associated with chemotherapy resistance, including resistance to 5‐FU and oxaliplatin.^[^ ^55^, ^56^, ^57^ ^]^ Among the downstream genes of the canonical Wnt pathway, we observed that ADAM10/NOTCH2/TCF7L2 signaling regulates MYC and JUN more prominently than the other genes. MYC and JUN, well‐known proto‐oncogenes, promote tumor growth and induce drug resistance.^[^ ^58^, ^59^, ^60^ ^]^ Our study revealed that ADAM10/NOTCH2 signaling promotes tumor growth and chemotherapy resistance by activating the transcription of MYC and JUN in CRC. In triple‐negative breast cancer, ADAM10 is implicated in oncogenic processes and chemoresistance by regulating the NOTCH1 signaling pathway.^[^ ^61^ ^]^ Many proteins were revealed as the substrates of ADAM10, such as NOTCH ligands (Dll1), Amyloid precursor protein (APP) and E‐cadherin, etc.^[^ ^62^ ^]^ Our results indicated that ADAM10 may tend to bind NOTCH2 in the context of colorectal organoids, and ADAM10 may interact with other NOTCHs in other types of cells or at certain conditions. The potential mechanisms are still to be explored in the further studies. In human breast cancer cells, the ADAM10 and ADAM17 inhibitor, INCB3619, along with a lapatinib‐like dual inhibitor of endothelial growth factor receptor and HER‐2/neu kinases, demonstrated synergistic growth inhibition.^[^ ^48^ ^]^ Aderbasib, another inhibitor of ADAM10 and ADAM17, overcomes endothelial growth factor receptor inhibitor resistance and enhance fluorouracil sensitivity in CRC.^[^ ^63^ ^]^ Simultaneously, using a CRC organoid model in vitro and in vivo, we observed that adavivint enhanced FOLFOX sensitivity by suppressing ADAM10/NOTCH2 signaling‐mediated regulation of Wnt target genes. Therefore, transcriptional activation of the Wnt pathway and high levels of ADAM10/NOTCH signaling are indications for adavivint treatment of CRC.

**Figure 8:**
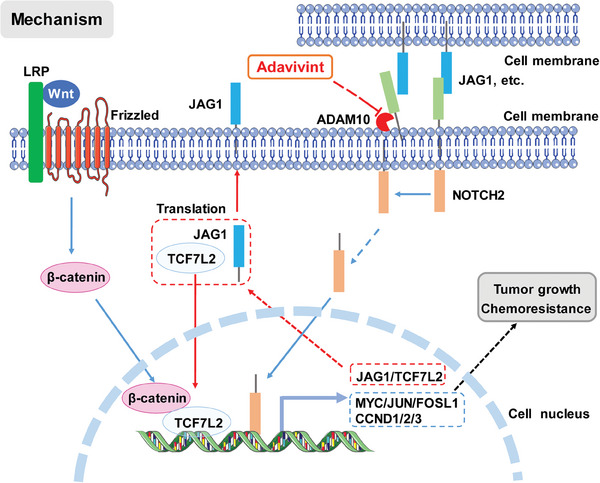
Mechanism of adavivint suppressing bypass ADAM10/NOTCH2/TCF7L2 signaling mediated transcriptional regulation of Wnt pathway to suppress tumor growth and overcome chemoresistance in CRC.

In conclusion, this study conducted integrated transcriptomic analysis of tumor organoids and tissues to assess the transcriptional regulation of the Wnt pathway and its association with liver metastasis in CRC. By screening inhibitors targeting the Wnt pathway in vitro and in vivo, we identified adavivint as a promising anticancer agent for CRC treatment. Mechanistically, adavivint suppressed ADAM10/NOTCH2/TCF7L2 signaling, which served as a bypass pathway to activate the transcription of Wnt target genes. Adavivint treatment and blockage of ADAM10/NOTCH2/TCF7L2 signaling effectively induced growth arrest and susceptibility to chemotherapy (**Figure** **8**). Overall, this study provides a promising anticancer agent and suggests a potential target for CRC treatment.

## 4 Experimental Section

### Organoid Culture

Eight primary CRC and paired normal colorectal tissues were obtained from the Shanghai East Hospital of Shanghai Tongji University, which was approved by the Institutional Review Board of the Shanghai East Hospital of Shanghai Tongji University. Eight CRC metastatic and four paired normal liver tissues were obtained from the Department of Hepatic Surgery, Fudan University Shanghai Cancer Center, which was approved by the Institutional Review Board of the Fudan University Shanghai Cancer Center. Two primary LUAD and two PDAC tissues were obtained from the Department of Thoracic Surgery, Ruijin Hospital of Shanghai Jiaotong University School of Medicine and Department of Hepatobiliary Surgery, Shanghai Sixth People's Hospital of Shanghai Jiaotong University School of Medicine, respectively. This study was approved by the Institutional Review Board of Ruijin Hospital of Shanghai Jiaotong University School of Medicine and Shanghai Sixth People's Hospital of Shanghai Jiaotong University School of Medicine. Organoid culture was performed as previously described.^[^ ^64^ ^]^ Written informed consent was obtained from all patients before tissue acquisition. Clinical characteristics of the patients were presented in Table S3 (Supporting Information). Culture media used for CRC organoids (WM‐H‐03), PDAC organoids (WM‐H‐05), LUAD organoids (WM‐H‐10), human colorectal organoid expansion (WM‐H‐01), and human colorectal differentiation (WM‐H‐01D) were purchased from Shanghai OuMel Biotechnology Co., Ltd. To explore whether CRC organoids grow independent of RSPO1, another organoid culture medium was prepared with advanced Dulbecco's modified Eagle's medium (DMEM)/F12 (Gbico), 100 µg mL^−1^ human RSPO1 (Sino Biological), 100 µg mL^−1^ human noggin (Sino Biological), 1 × B27 (Gbico), 1.25 mM n‐acetyl cysteine (MCE), 10 mM nicotinamide (MCE), 60 ng/mL human EGF (Sino Biological), 100 ng mL^−1^ human FGF10 (Sino Biological), 10 nM gastrin (MCE), 500 nM A83‐01 (MCE), 3 µM SB202190 (MCE), 20 nM prostaglandin E2 (MCE), and 100 µg mL^−1^ primocin (InvivoGen).

### Whole Exome Sequencing Analysis

After end‐repairing, A‐tailing, and ligation, the fragmented genomic DNA was sequentially mixed with indexed adapters, followed by size selection using the Agencourt AMPure XP beads (Beckman Coulter Inc., Brea, CA, USA). Library was constructed using the KAPA Library Preparation kit (Kapa Biosystems, Inc., Wilmington, MA, USA), and PCR (7–8 cycles according to the amount of DNA used) was performed with pre‐capture ligation‐mediated PCR (Pre‐LM PCR) Oligos (Kapa Biosystems, Inc.) in 50‐µL reaction mixtures. DNA sequencing was performed on an Illumina NovaSeq 6000 system, according to the manufacturer's instructions, at an average depth of 300×. Sequencing data were aligned to the hg38 genome (GRch38) using the Burrows‐Wheeler Aligner. SAMtools was used to sort the BAM files and perform duplicate markings. After removing the duplicate reads using Gencore version 0.12.0 (https://github.com/OpenGene/gencore), single nucleotide variants and insertions/deletions were detected in tumor and matched‐normal pairs using Mutect2 from GATK. MutSigCV (version 1.41) was used to determine the significantly mutated genes with q‐value < 0.05.

### RNA‐Seq Analysis

Fresh tissues (normal colorectal tissues: n = 7; primary CRC tissues: n = 7; normal liver tissue: n = 4; liver‐metastatic CRC tissues: n = 9) and cultured organoids (colorectal organoid: n = 4; primary CRC organoids: n = 6; liver‐metastatic organoids: n = 9) were used for RNA‐seq. After treatment with adavivint for 24 h or siRNA for 96 h, the organoids were harvested for RNA‐seq. Library construction was performed using the TruSeq RNA Sample Preparation Kit (Illumina), and RNA sequencing was performed using Illumina HiSeq Xten (Illumina). After quality control, the gene counts were normalized to transcripts per million (TPM) and log2(TPM + 1)‐transformed expression data for further analysis. Finally, 189 gene sets of KEGG pathways were downloaded from GSEA database (https://www.gsea‐msigdb.org/gsea/index.jsp), and enrichment scores of these gene sets were calculated using the “GSVA” package in R software. GSVA, a single‐sample gene set enrichment method, was performed to estimate the KEGG pathway activity by transforming an input gene‐by‐sample expression data matrix into a corresponding gene‐set‐by‐sample expression data matrix. The resulting expression data matrix was used with classical analytical methods to assess the differential expression between normal and tumor groups. After calculating the enrichment scores of KEGG pathways, difference analysis was performed between two groups. All RNA‐seq data used in this study were shown in File S1 (Supporting Information).

### Transfection of Organoids

Serum‐free DMEM containing 2% basement membrane component (OM‐MG‐02; OuMel Biotechnology Co., Ltd.) was plated in a 24‐well plate (400 µL well^−1^) and incubated at 37 °C for 2 h. Organoids were dispersed in a dissociation solution (OM‐D‐01; OuMel Biotechnology Co., Ltd.), and the number of dissociated cells was calculated. After removing the medium from the 24‐well plate, the organoid medium containing 10^5^ cells was plated in a 24‐well plate for subsequent experiments. On the second day, the medium was removed and 500 µL serum‐free DMEM containing siRNA or plasmid wrapped by Lipofectamine 2000 (Invitrogen, Carlsbad, CA, USA) was added. After culturing for 24 h, the medium was exchanged with the organoid culture. The plasmids and siRNAs used in this study were purchased from GeneChem (Shanghai, China) and GenePharma (Shanghai, China), respectively. All siRNA sequences were listed in Table S4 (Supporting Information). The lentivirus containing sgRNA targeting TCF7L2 or NOTCH2 was purchased from GeneChem. All sgRNA sequences were listed in Table S4 (Supporting Information). The most effective siRNA or sgRNA targeting TCF7L2 or NOTCH2 and the most effective sequences were used for subsequent experiments.

### High‐Throughput Screening of Wnt Pathway‐Targeting Inhibitors Using CRC Organoids

Organoids in good conditions (passages 3–8) were dispersed in a dissociation solution. Then, 4 µL mixture of basement membrane components and DMEM (1:1) containing 2 × 10^3^ cancer cells was placed in every well of a 96‐well plate. On the third day, the 1 µM concentrations of compounds were added, and the organoids were cultured for another four days. For the FOLFOX regimen, the concentration ratio of 5‐fluorouracil, calcium folinate, and oxaliplatin was 25:5:1. On the seventh day, the medium was removed, and a fresh medium containing 10% cell counting kit‐8 was added. After incubation for 3 h, the absorbance (OD) was measured at 450 nm by enzyme marker.

### Cell Proliferation Assay

Cells transfected with organoids were dispersed in a dissociation solution (OM‐D‐01; OuMel Biotechnology Co., Ltd.). Then, 4 µL mixture of basement membrane components (OM‐MG‐02; OuMel Biotechnology Co., Ltd.) and DMEM (1:1) containing 2 × 10^3^ cancer cells was placed in every well of a 96‐well plate. On the first, third, and fifth days, absorbance (OD) values were measured at 450 nm using the cell counting kit‐8 kit was purchased from (DOJINDO, CK04, Kumamoto, Japan). To assess and quantify the morphological parameters, brightfield images from four independent wells were captured (40×), and organoid number and mean organoid area (π× radius [µm] × radius [µm]) were calculated as previously described.^[^ ^65^ ^]^

### Western Blotting and qRT‐PCR

As the organoids grew to the desired number, total protein and total RNA were extracted using the TRIzol solution (15596‐026; Invitrogen) and lysis buffer (P0013B; Beyotime, Beijing, China), and western blotting and qRT‐PCR assays were performed as previously described.^[^ ^64^ ^]^ The working dilutions of antibodies used here were listed in Table S5 (Supporting Information). Reverse transcription kit (FSQ‐101; TOYOBO, Osaka, Japan) and SYBR Select Master Mix (4472908; Applied Biosystems, Foster City, USA) were used for qRT‐PCR in a 10‐µL reaction mixture with HT 7900 (Applied Biosystems). All primer sequences were listed in Table S6 (Supporting Information).

### HE Staining, IHC, and Immunofluorescence (IF) Analysis

Tissue sections were prepared, and HE staining, IHC, and IF analysis were conducted as described in this previous report.^[^ ^64^ ^]^ All working dilutions of antibodies used here were listed in Table S5 (Supporting Information). IHC was used to quantify the expression levels of ADAM10 and NOTCH2 as described in this previous study.^[^ ^64^ ^]^ For IF experiments, the mean fluorescence intensity of triplicate experiments was calculated using the ImageJ software.

### IP Assay

As the organoids grew to the desired number, basement membrane components were removed by adding phosphate‐buffered saline containing 10 mM EDTA. Organoids were harvested using an IP lysis buffer (P0013; Beyotime). Antibodies against ADAM10, TCF7L2, and TCF7L2 and magnetic beads (20 µL; 88802; Thermo Scientific, MS, USA) were added to the lysate and incubated overnight at 4 °C. After washing thrice with the lysis buffer, western blotting was performed to analyze the precipitates. The antibody concentrations were presented in Table S5 (Supporting Information).

### Proteome Analysis Using Liquid Chromatography‐Tandem MS (LC‐MS/MS)

After 24‐h adavivint treatment, the total protein was extracted and quantified via high‐resolution MS using the MaxQuant and Perseus software to screen the differentially expressed proteins and subsequently perform bioinformatic analysis. Briefly, 50 µg of protein was mixed with 50 mM ammonium bicarbonate solution to make up the volume to 50 µL. Then, dithiothreitol was added to make the final concentration 10 mM and incubated at 37 °C for 60 min. Iodoacetamide was added to a final concentration of 50 mM and allowed to react for 30 min in the dark at room temperature. The solution was transferred to an ultrafiltration tube for high‐speed centrifugation and washed twice with 50 mM ammonium bicarbonate (containing 0.8% SDC). Trypsin was added and hydrolyzed in a 37 °C incubator for 12–16 h. The enzymatic hydrolysates were washed and pooled with 50 mM ammonium bicarbonate. TFA was added for acidification, and the mixture was centrifuged to remove SDC and desalted using the C18 desalting column. Finally, the desalted samples were lyophilized and resolved using 0.1% FA for MS analysis.

### Cleavage Under Targets and Tagmentation (Cut&Tag) Assay for RNA‐Seq and PCR Analyses

Organoids with good growth were digested to obtain a single cell suspension. After counting the cells, 5 × 10^5^ cells were used for the subsequent assay. Cut&tag assay was performed using the Hyperactive Universal CUT & Tag Assay Kit for Illumina (TD903; Vazyme, Nanjing), according to the manufacturer's instructions. Sequencing libraries were prepared using the TruePrep Index Kit V2 (TD202; Vazyme), according to the manufacturer's instructions. The PCR products were cleaned using clean VAHTS DNA beads (N411‐01‐AA; Vazyme). RNA sequencing was performed using Illumina HiSeq Xten (Illumina) at the HaploX Genomics Center (Shanghai, Jiangxi, China). The Trim_galore package was used to remove the adaptors, and the trimmed reads were aligned to the hg38 reference genome using Bowtie2. Narrow peaks were called using the MACS2 package, and IGV files were produced using bamCoverage of deepTools for visualization in IGV 2.16.0. Finally, peak annotation and motif finding were performed using the Homer software. Simultaneously, the immunoprecipitated DNA was harvested and analyzed using PCR. Specific primers for TCF7L2 promoter designed according to peak calling and annotation analysis were listed in Table S6 (Supporting Information).

### Establishment of FOLFOX‐Resistant Organoids

FOLFOX‐sensitive organoids (P2 and LM5R) were selected to construct the drug‐resistant strains. When the growth was optimum, these organoids were treated with FOLFOX containing 10 µM 5‐fluorouracil for two days (concentration ratio of 5‐fluorouracil, calcium folinate, and oxaliplatin = 25:5:1). The culture medium was replaced every two days until the organoids reached a certain count. Then, these organoids were treated with a low‐dose FOLFOX regimen (1 µM 5‐fluorouracil). When the organoids reached a certain count, the sensitivity to FOLFOX was tested. If the IC50 of the resistant organoids was five‐times greater than that of the parental organoids, the establishment of FOLFOX‐resistant organoids was considered successful.

### PDOX Model

Animal experiments were approved by the Institutional Animal Care and Use Committee of Fudan University (FUSCC‐IACUC‐S20210285). As the organoids grew to the desired number, the dome‐containing organoids and organoid medium were fully mixed and transferred to centrifugal tubes. After centrifugation (800 rpm) for 5 min, the medium was removed, and the organoids were re‐suspended in serum‐free DMEM. To count the cells of organoids per mL, 50 µL medium containing organoids was used for dissociation and calculation. Then, 100 µL mixture of organoids (5 × 10^5^) and basement membrane components (1:1) was inoculated subcutaneously. When the subcutaneous tumor grew to 3–4 mm in diameter, drugs were administered. Adavivint (5 mg/kg or 50 mg/kg) was intraperitoneally injected daily, and FOLFOX (5‐fluorouracil/calcium folinate/oxaliplatin = 50/39/6 mg/kg) was intraperitoneally administered twice in one week. The tumor size was measured once a week and calculated as width × width × length/2.

### Data Extraction and Analysis

The gene count data of 635 CRC cases and APC mutation data of 533 CRC cases was downloaded from TCGA database (http://cancergenome.nih.gov/). Gene counts were normalized to TPM, and log2(TPM + 1)‐transformed expression data were used for further analysis. Finally, 189 gene sets of KEGG pathways were downloaded from GSEA database (https://www.gsea‐msigdb.org/gsea/index.jsp), and enrichment scores of these gene sets were calculated using the “GSVA” package in R software.

### Statistical Analyses

Bubble diagram was plotted by using “ggplot2” and “ggsave” package in R software. Student's *t*‐test was performed using the GraphPad Prism 6.0 software. A histogram was used to observe the‐Log10P values determined in Gene Ontology analysis. Heatmap of spearman correlation values of organoids based on RNA‐seq data using 10000 most variable genes were plotted using the “ggplot2” and “data.table” packages in R software. Pearson correlation between two variables was analyzed using the “corrplot” and “PerformanceAnalytics” packages in R software or GraphPad Prism 6.0. Heatmap and Volcano plot were plotted using the “pheatmap” and “ggplot2” packages in R software, respectively. Gene mutation and copy number were determined using the “ComplexHeatmap” package in R. Tumor growth curves and dose–response curves were plotted, and AUC values was calculated using GraphPad Prism 6.0. Statistical significance was set at P< 0.05. All results were represented as the mean ± standard deviation of three experiments.

## Conflict of Interest

The authors declare no conflict of interest.

## Author Contributions

L.W., G.X., Y.Z., and W.X. conceived the project. Z.X., Y.Z., Y.W., and X.M. designed the experiments. Z.X., Y.W., L.F., B.Y., and Y.W. conducted experiments, such as Cut&Tag, RNA‐seq, IHC, Western blot, CoIP, etc. Z.X., S.S., and J.Z. analyzed the data. Z.X., S.Y. and Y.H. wrote the manuscript. Y.Z. and G.X. reviewed and polished the manuscript.

## Supporting information



Supporting Information

Supplementary File S1

## Data Availability

The data that support the findings of this study are available in the supplementary material of this article.
