# Supplementary material for: Targeting the NOTCH2/ADAM10/TCF7L2 Axis‐Mediated Transcriptional Regulation of Wnt Pathway Suppresses Tumor Growth and Enhances Chemosensitivity in Colorectal Cancer
Source: Adv Sci (Weinh). 2024 Nov 27;12(3):2405758. doi: 10.1002/advs.202405758 (PMC11744699; doi:10.1002/advs.202405758)
Supplement: Supplementary file 1 — Supporting Information [file ADVS-12-2405758-s001.docx]

**Supplementary Figures**


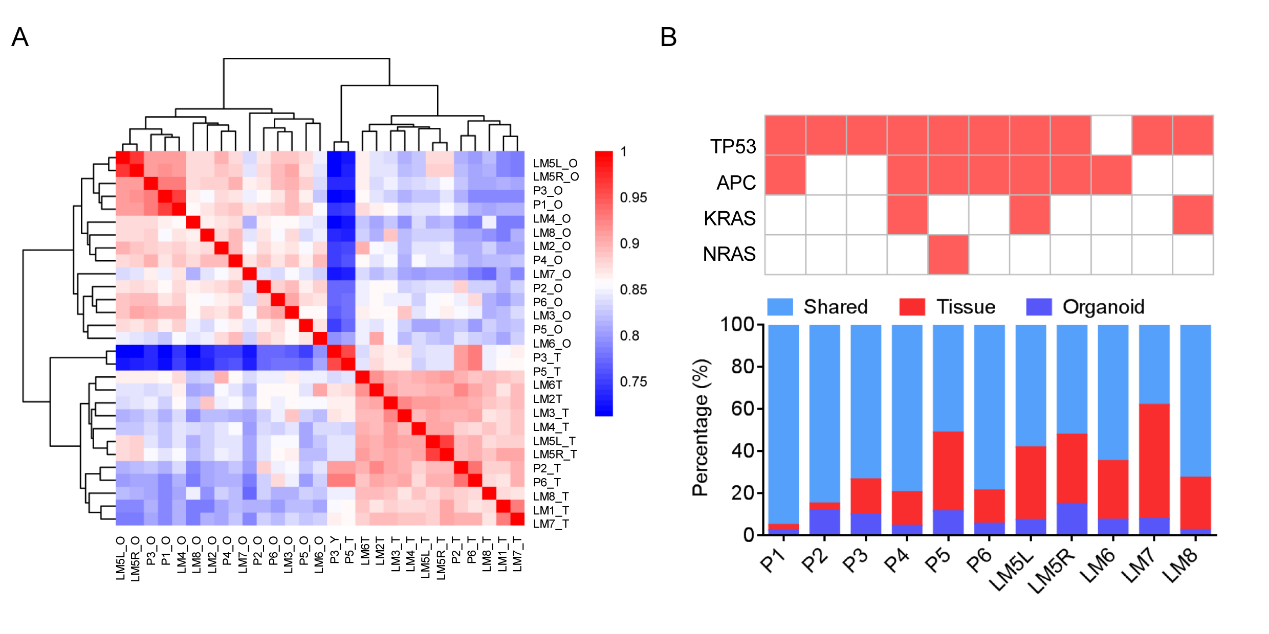


Figure S1. Verification of CRC organoids by RNA and WES sequencing. A) Heatmap of spearman correlation values of organoids based on RNA-seq data using 10000 most variable genes, and clustering method of “complete” was utilized. Suffix “O” and suffix “T” represent tumor organoid and tumor tissue respectively. B) Bar plots indicate the intersected gene mutations between organoids and corresponding tumor tissues (below), and the somatic mutations of TP53, APC, KRAS and NRAS found in tumor organoids and corresponding tumor tissues were also displayed (above).


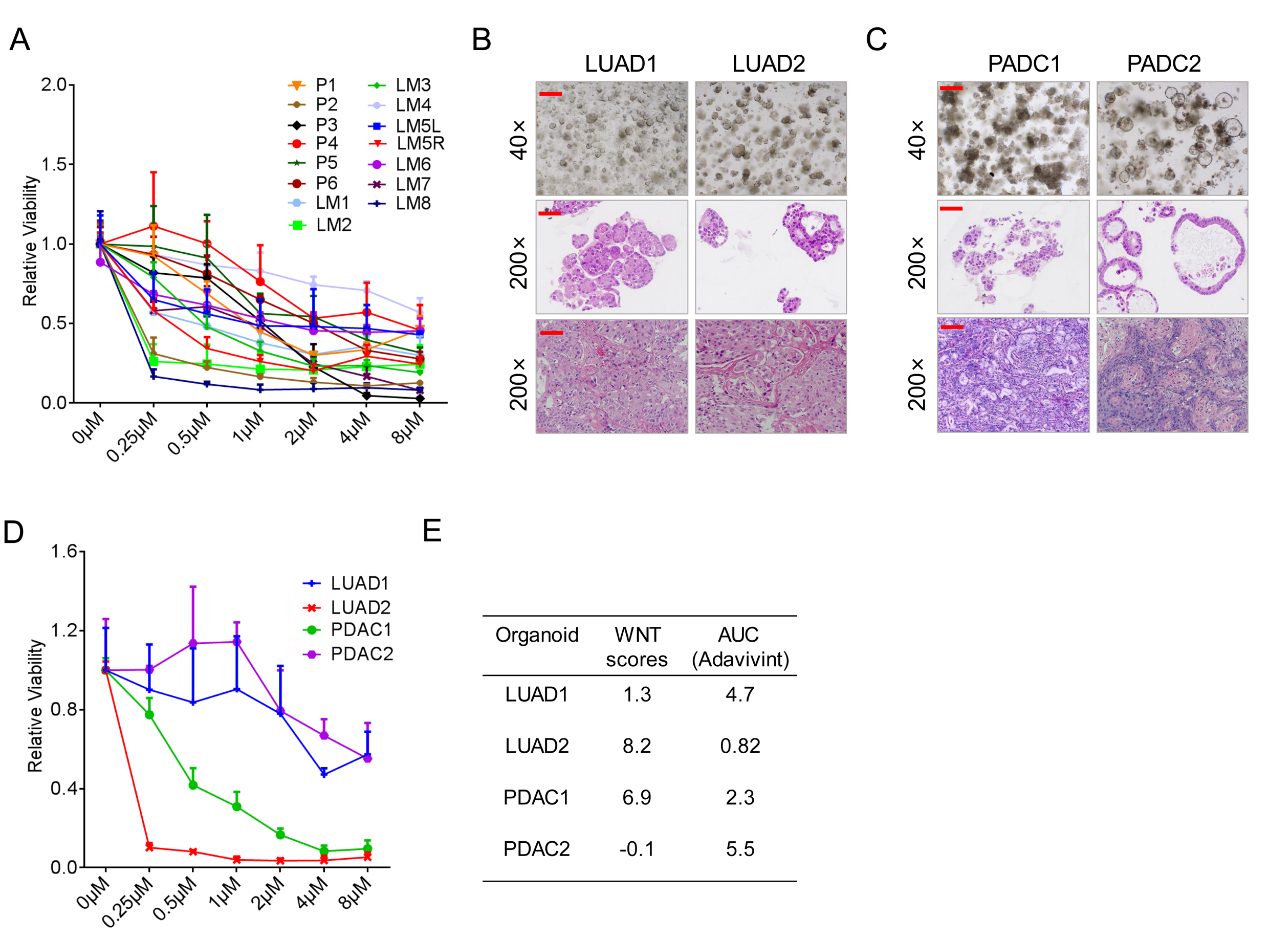


Figure S2. Adavivint sensitivity of CRC, LUAD and PDAC organoids by CCK8 assay. A) Dose-response curves for adavivint in 6 primary CRC organoids and 9 liver-metastatic CRC organoids. Representative bright-field microscopy images of organoids derived from primary tumor tissues, and HE staining of these organoids and corresponding tissues (B, NSCLC, n=2; C, PDAC, n=2). D) Dose-response curves for adavivint in 2 LUAD organoids and 2 PDAC organoids, and E) WNT scores and AUC values of adavivint are listed. Bar: 40×, 200μm; 200×, 50μm.


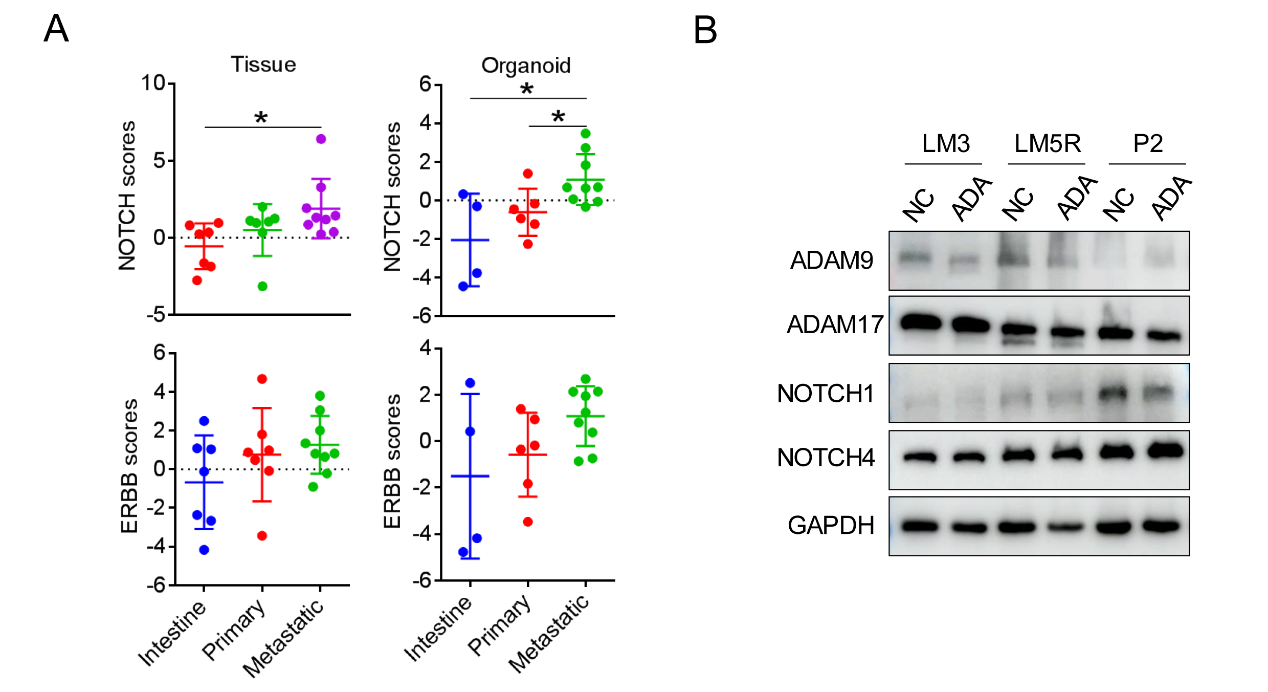


Figure S3. A) Expression of ERBB and NOTCH pathway enrichment scores in normal colorectal (n=7), primary (n=7), metastatic CRC (n=9) tissues, and normal colorectal (n=4), primary (n=6) and metastatic CRC (n=9) tissues-derived organoids. B) Members of ADAM and NOTCH family are detected by Western blot after treatment of adavivint for 24 hours.


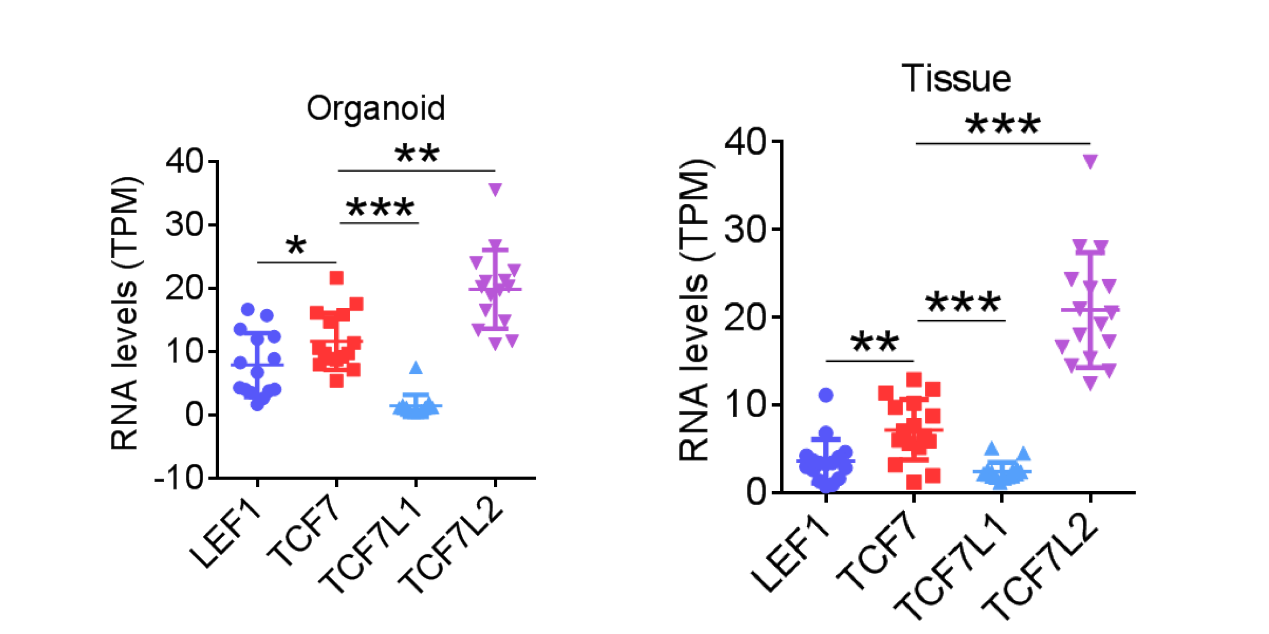


Figure S4. Bar plots indicate the RNA levels of LEF1, TCF7, TCF7L1 and TCF7L2 by TPM transformation using RNA sequencing data in tumor tissues (n=16) and organoids (n=15). All P-values in are calculated using an unpaired two-sided Student’s t-test, and data presented as mean ± SD. *P<0.05, **P<0.01, ***P<0.001.


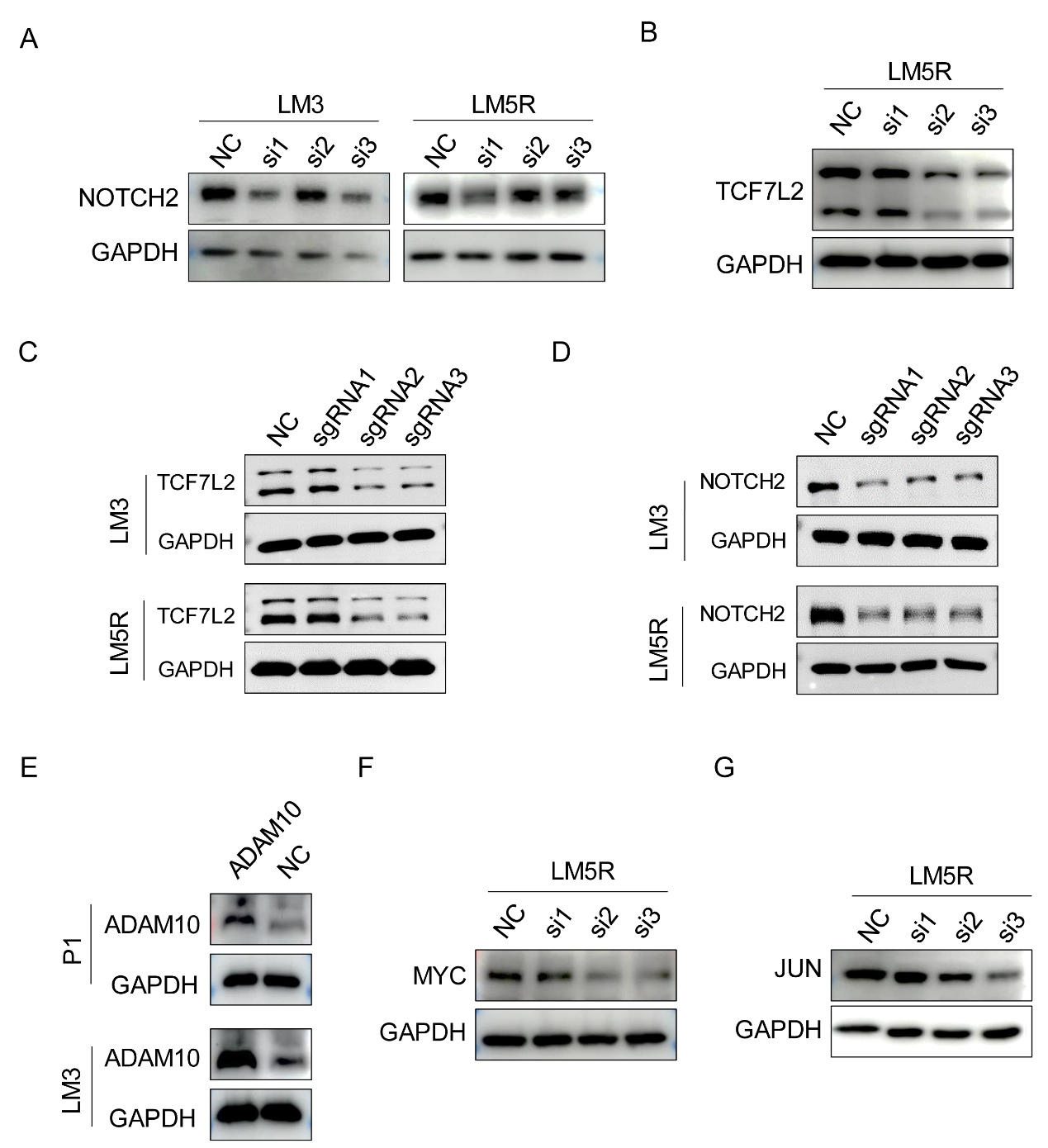


Figure S5. Knockdown of NOTCH2 (A), TCF7L2 (B), MYC (F) and JUN (G) by siRNA transfection, forcing expression of ADAM10 (E) by plasmid transfection, and stable knockdown using lentivirus containing sgRNA targeting TCF7L2 (C) or NOTCH2 (D) in the indicated CRC organoids.


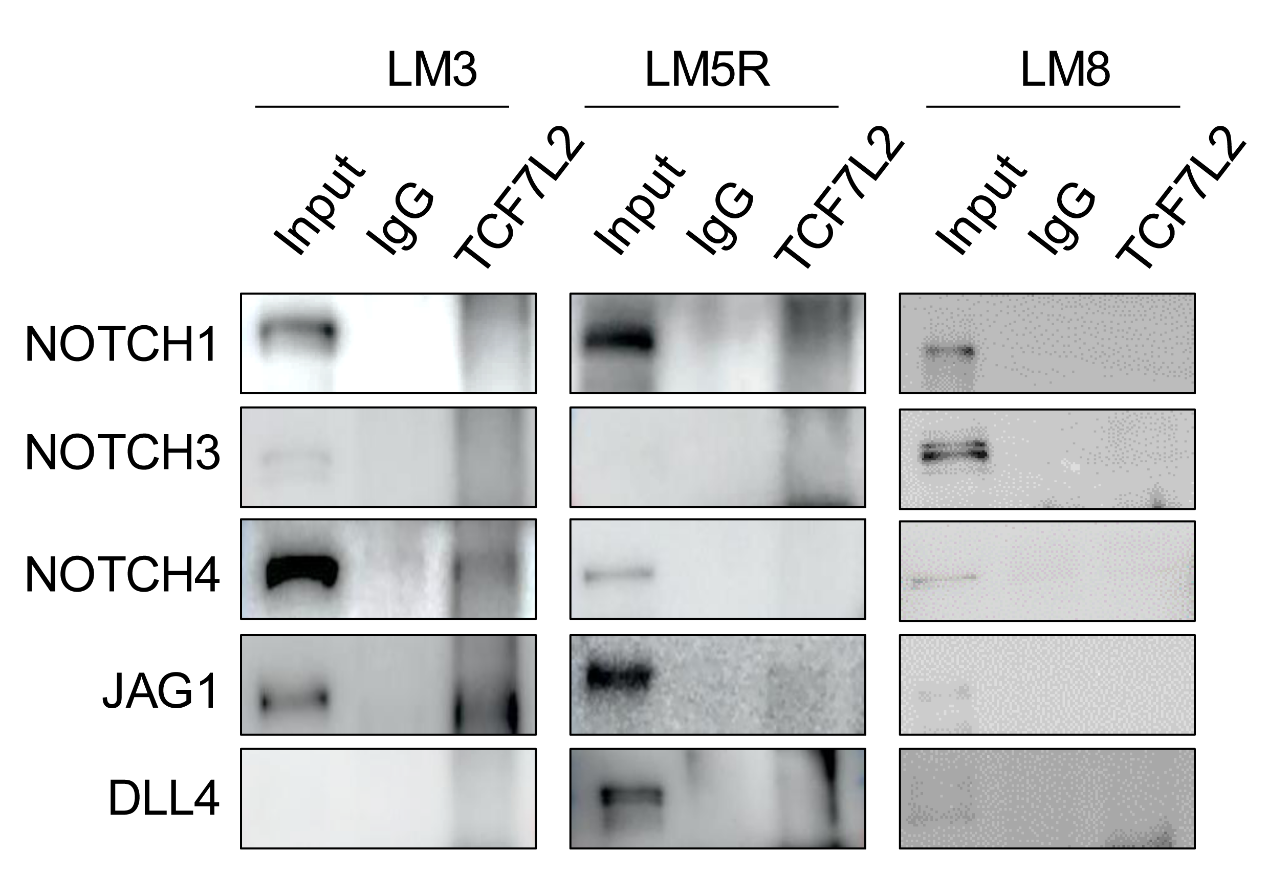


Figure S6. Immunoprecipitation assay are conducted to exclude direct interactions of TCF7L2 with members of NOTCH pathway in LM3, LM5R and LM8 organoids.


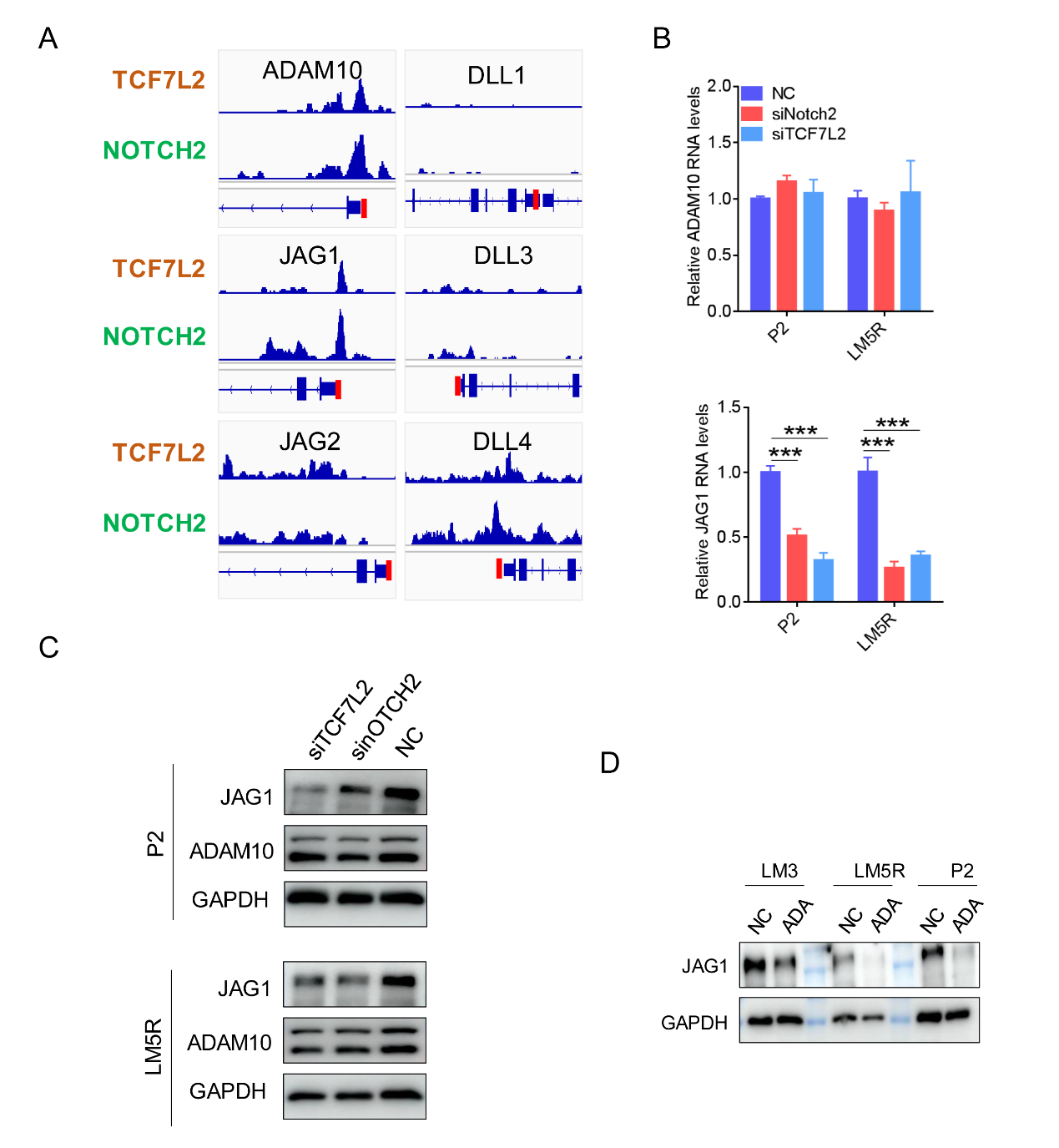


Figure S7. Both NOTCH2 and TCF7L2 directly promote transcription of Notch ligand JAG2, and adavivint treatment attenuates expression of Notch ligand JAG2 in CRC organoids. A) Cut&tag assay shows that TCF7L2 and NOTCH2 could be enriched in promoters of ADAM10 and NOTCHs ligands. The red bar indicates gene start site. After NOTCH2 or TCF7L2 knockdown in CRC organoid, B) qRT-PCR and C) Western blot are used to examine expression of ADAM10 and JAG1. All P-values in are calculated using an unpaired two-sided Student’s t-test, and data presented as mean ± SD. ***P<0.001.D) Notch ligand JAG1 are detected by Western blot after treatment of adavivint for 24 hours. ADA indicates “adavivint”.


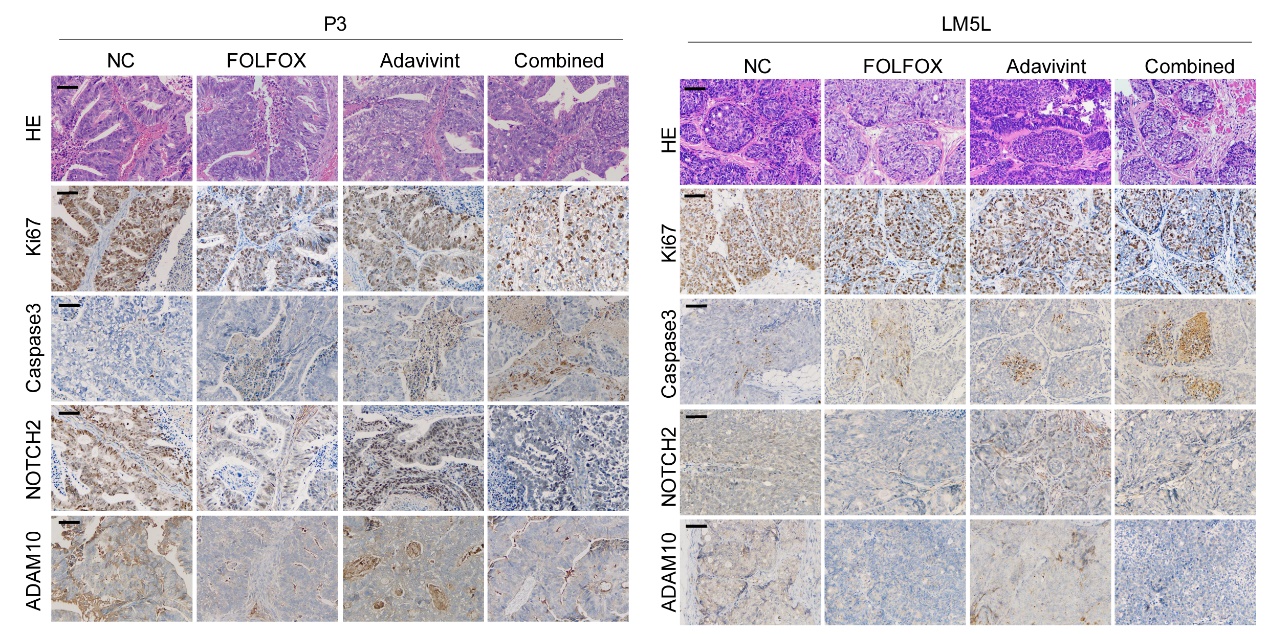


Figure S8. Expression of ADAM10, NOTCH2, Ki67 and Caspase3 by IHC analysis in the xenograft tumor established using the indicated CRC organoids and treated by adavivint or/and FOLFOX regimen. Bar: 200×, 50μm.

**Supplementary tables**

Table S1. Compounds and chemicals used in this study.

| Compound/chemical | Company | Catalog |
| --- | --- | --- |
| CWP232228 | MCE | HY-18959 |
| DK419 | MCE | HY-112799 |
| FIDAS-3 | MCE | HY-136145 |
| Fz7-21 (TFA) | MCE | HY-P1454A |
| Gigantol | MCE | HY-N2523 |
| iCRT 14 | MCE | HY-16665 |
| IWP-4 | MCE | HY-12879 |
| IWR-1 | MCE | HY-12238 |
| JW74 | MCE | HY-19739 |
| KY02111 | MCE | HY-13815 |
| KY1220 | MCE | HY-102028 |
| KYA1797K | MCE | HY-101090 |
| LF3 | MCE | HY-101486 |
| MSAB | MCE | HY-120697 |
| Nefopam (hydrochloride) | MCE | HY-B1057 |
| NRX-252262 | MCE | HY-111760 |
| Pamidronic acid | MCE | HY-B0012 |
| Tegatrabetan | MCE | HY-109103 |
| Teplinovivint | MCE | HY-137454 |
| ZW4864 (free base) | MCE | HY-132300A |
| β-catenin-IN-2 | MCE | HY-136464 |
| n-Acetyl Cysteine | MCE | HY-B0215 |
| Nicotinamide | MCE | HY-B0150 |
| Gastrin | MCE | HY-P1097 |
| A83-01 | MCE | HY-10432 |
| SB202190 | MCE | HY-10295 |
| Prostaglandine | MCE | HY-101952 |

Table S2. Correlation analysis between ADAM10 mRNA RNA level and enrichment scores of KEGG pathways in CRC organoid.

| Pathway | r | P (two-tailed) |
| --- | --- | --- |
| KEGG_GAP_JUNCTION | 0.8262 | 0.0001 |
| KEGG_UBIQUITIN_MEDIATED_PROTEOLYSIS | 0.8157 | 0.0002 |
| KEGG_SMALL_CELL_LUNG_CANCER | 0.8128 | 0.0002 |
| KEGG_NON_SMALL_CELL_LUNG_CANCER | 0.7961 | 0.0004 |
| KEGG_GLIOMA | 0.7957 | 0.0004 |
| KEGG_TYPE_II_DIABETES_MELLITUS | 0.7838 | 0.0005 |
| KEGG_NOTCH_SIGNALING_PATHWAY | 0.77 | 0.0008 |
| KEGG_ENDOMETRIAL_CANCER | 0.7671 | 0.0008 |
| KEGG_ERBB_SIGNALING_PATHWAY | 0.7635 | 0.0009 |
| KEGG_COLORECTAL_CANCER | 0.7623 | 0.001 |
| KEGG_PROSTATE_CANCER | 0.7621 | 0.001 |
| KEGG_HIPPO_ SIGNALING_PATHWAY | 0.7604 | 0.001 |
| KEGG_BASAL_CELL_CARCINOMA | 0.7594 | 0.001 |
| KEGG_CHRONIC_MYELOID_LEUKEMIA | 0.7592 | 0.001 |
| KEGG_MELANOMA | 0.7556 | 0.0011 |
| KEGG_BASAL_TRANSCRIPTION_FACTORS | 0.7552 | 0.0011 |
| KEGG_MELANOGENESIS | 0.7508 | 0.0013 |
| KEGG_TGF_BETA_SIGNALING_PATHWAY | 0.7467 | 0.0014 |
| KEGG_THYROID_CANCER | 0.7334 | 0.0019 |
| KEGG_HEDGEHOG_SIGNALING_PATHWAY | 0.7308 | 0.002 |
| KEGG_NEUROTROPHIN_SIGNALING_PATHWAY | 0.7306 | 0.002 |
| KEGG_LYSINE_DEGRADATION | 0.7142 | 0.0028 |
| KEGG_PROGESTERONE_MEDIATED_OOCYTE_MATURATION | 0.7134 | 0.0028 |
| KEGG_RENAL_CELL_CARCINOMA | 0.7121 | 0.0029 |
| KEGG_MTOR_SIGNALING_PATHWAY | 0.7065 | 0.0032 |
| KEGG_T_CELL_RECEPTOR_SIGNALING_PATHWAY | 0.6974 | 0.0039 |
| KEGG_INSULIN_SIGNALING_PATHWAY | 0.6962 | 0.0039 |
| KEGG_PATHWAYS_IN_CANCER | 0.6918 | 0.0043 |
| KEGG_WNT_SIGNALING_PATHWAY | 0.6888 | 0.0045 |
| KEGG_WNT_SIGNALING_PATHWAY | 0.6888 | 0.0045 |
| KEGG_CIRCADIAN_RHYTHM_MAMMAL | 0.6813 | 0.0052 |
| KEGG_MAPK_SIGNALING_PATHWAY | 0.6656 | 0.0068 |
| KEGG_AXON_GUIDANCE | 0.6651 | 0.0068 |
| KEGG_VASCULAR_SMOOTH_MUSCLE_CONTRACTION | 0.6608 | 0.0073 |
| KEGG_DORSO_VENTRAL_AXIS_FORMATION | 0.6587 | 0.0076 |
| KEGG_VEGF_SIGNALING_PATHWAY | 0.645 | 0.0094 |
| KEGG_LONG_TERM_POTENTIATION | 0.6429 | 0.0097 |
| KEGG_INOSITOL_PHOSPHATE_METABOLISM | 0.6413 | 0.01 |
| KEGG_ALDOSTERONE_REGULATED_SODIUM_REABSORPTION | 0.6382 | 0.0105 |
| KEGG_REGULATION_OF_ACTIN_CYTOSKELETON | 0.6168 | 0.0143 |
| KEGG_RNA_DEGRADATION | 0.6142 | 0.0149 |
| KEGG_ACUTE_MYELOID_LEUKEMIA | 0.6048 | 0.0169 |
| KEGG_CALCIUM_SIGNALING_PATHWAY | 0.6036 | 0.0172 |
| KEGG_LONG_TERM_DEPRESSION | 0.5931 | 0.0198 |
| KEGG_PHOSPHATIDYLINOSITOL_SIGNALING_SYSTEM | 0.5878 | 0.0212 |
| KEGG_PANCREATIC_CANCER | 0.5775 | 0.0242 |
| KEGG_RAP1_ SIGNALING_PATHWAY | 0.5749 | 0.025 |
| KEGG_NOD_LIKE_RECEPTOR_SIGNALING_PATHWAY | 0.569 | 0.0269 |
| KEGG_OOCYTE_MEIOSIS | 0.5648 | 0.0283 |
| KEGG_FOCAL_ADHESION | 0.5567 | 0.0311 |
| KEGG_GNRH_SIGNALING_PATHWAY | 0.5542 | 0.032 |
| KEGG_ONE_CARBON_POOL_BY_FOLATE | 0.5335 | 0.0406 |
| KEGG_CELL_CYCLE | 0.5329 | 0.0408 |
| KEGG_ADHERENS_JUNCTION | 0.5291 | 0.0426 |
| KEGG_FC_GAMMA_R_MEDIATED_PHAGOCYTOSIS | 0.5251 | 0.0445 |
| KEGG_NUCLEOTIDE_EXCISION_REPAIR | 0.5248 | 0.0446 |
| KEGG_GLYCOSPHINGOLIPID_BIOSYNTHESIS_GLOBO_SERIES | -0.5159 | 0.049 |

Table S3. Clinical information of CRC, LUAD and PDAC used for organoid establishment in this study.

| ID | Gender | Age (years) | Pathology | Differentiation | Organoid  establishment | Diagnostic exon sequencing |
| --- | --- | --- | --- | --- | --- | --- |
| P1 | Female | 69 | Adenocarcinoma | Intermediate | Yes | NA |
| P2 | Male | 46 | Adenocarcinoma | Intermediate | Yes | NA |
| P3 | Female | 78 | Adenocarcinoma | Intermediate | Yes | NA |
| P4 | Male | 76 | Adenocarcinoma | Intermediate | Yes | NA |
| P5 | Male | 69 | Adenocarcinoma | Intermediate/Low | Yes | NA |
| P6 | Male | 54 | Adenocarcinoma | Intermediate | Yes | NA |
| P7 | Male | 58 | Adenocarcinoma | Intermediate | Yes | NA |
| LM1 | Male | 69 | Adenocarcinoma | Intermediate/Low | Yes | APC+/TP53+ |
| LM2 | Female | 62 | Adenocarcinoma | Intermediate | Yes | NA |
| LM3 | Female | 60 | Adenocarcinoma | Low | Yes | APC+/TP53+ |
| LM4 | Male | 63 | Adenocarcinoma | Intermediate/Low | Yes | NA |
| LM5 | Male | 49 | Adenocarcinoma | Intermediate/Low | Yes | NA |
| LM6 | Female | 63 | Adenocarcinoma | Intermediate | Yes | APC+/TP53+ |
| LM7 | Female | 44 | Adenocarcinoma | Intermediate/Low | Yes | APC+/TP53- |
| LM8 | Male | 55 | Adenocarcinoma | Intermediate/Low | Yes | APC-/TP53+ |
| LUAD1 | Male | 44 | Adenocarcinoma | NA | Yes | NA |
| LUAD2 | Female | 52 | Adenocarcinoma | NA | Yes | NA |
| PDAC1 | Male | 75 | Adenocarcinoma | Intermediate/Low | Yes | NA |
| PDAC2 | Male | 68 | Adenocarcinoma | Intermediate/Low | Yes | NA |

Table S4. SiRNA sequences used in this study.

| Gene (siRNA secquence) | Sense（5'-3'） | Antisense（5'-3'） |
| --- | --- | --- |
| ADAM10 | | |
| si-1 | GCCCAUGGAAGACAUUUCATT | UGAAAUGUCUUCCAUGGGCTT |
| si-2 | CCAGCAGAGAGAUAUAUUATT | UAAUAUAUCUCUCUGCUGGTT |
| si-3 | GUGCAUUAAUGGGCAAUGUTT | ACAUUGCCCAUUAAUGCACTT |
| NOTCH2 | | |
| si-1 | GCACCUGUGAGAGGAAUAUTT | AUAUUCCUCUCACAGGUGCTT |
| si-2 | GGCAGUGUGUGGAUAAAGUTT | ACUUUAUCCACACACUGCCTT |
| si-3 | GGAGGUCUCAGUGGAUAUATT | UAUAUCCACUGAGACCUCCTT |
| TCF7L2 | | |
| si-1 | GAACCUAUCUCCAGAUGAATT | UUCAUCUGGAGAUAGGUUCTT |
| si-2 | CACACAUUGUCUCUAACAATT | UUGUUAGAGACAAUGUGUGTT |
| si-3 | GACAGCUUCAUAUGCAACUTT | AGUUGCAUAUGAAGCUGUCTT |
| MYC | | |
| si-1 | GGAACUAUGACCUCGACUATT | UAGUCGAGGUCAUAGUUCCTT |
| si-2 | CCACACAUCAGCACAACUATT | UAGUUGUGCUGAUGUGUGGTT |
| si-3 | GAACACACAACGUCUUGGATT | UCCAAGACGUUGUGUGUUCTT |
| JUN | | |
| si-1 | CGAGUCGACAAGUAAGAGUTT | ACUCUUACUUGUCGACUCGTT |
| si-2 | GUGCGCUCUUAGAGAAACUTT | AGUUUCUCUAAGAGCGCACTT |
| si-3 | GUGACGGACUGUUCUAUGATT | UCAUAGAACAGUCCGUCACTT |
| TCF7L2 | | |
| sgRNA1 | AGCAATGAACACTTCACGCC | |
| sgRNA2 | AATACGGGGATATATCTGGA | |
| sgRNA3 | TGGTGAGGGTGCTGCACCAC | |
| NOTCH2 | | |
| sgRNA1 | CTACCAGTGCCAGTGCCCTC | |
| sgRNA2 | AACCACAGGTGTCAGAATGG | |
| sgRNA3 | ACCTGTGCCAACCGCAATGG | |

Table S5. Antibodies used in this study.

| Antibody | Company | Application and density |
| --- | --- | --- |
| Hu ADAM10 Rabbit mAb | CST (14194) , Boston, USA | WB (1:1000) |
| Hu ADAM10 Mouse mAb | Santa Cruz (sc-48400), California, USA | IHC (1:50)  IHC (1:50)  IF (1:50) |
| Anti -Caspase-3 Rabbit pAb | Servicebio (GB11767C), Wuhan, China | IHC (1:50) |
| HRP-Conjugated GAPDH Antibody | proteintech (HRP-60004), Wuhan, China | WB (1:3000) |
| Donkey anti-Rabbit IgG (H+L) | Invivogen (A-21206), California, USA | IF (1:200) |
| ADAM9 (D64B5) Rabbit mAb | CST (4151) , Boston, USA | WB (1:1000) |
| DLL1 Antibody | CST (2588) , Boston, USA | WB (1:1000) |
| DLL3 (G93) Antibody | CST (2483) , Boston, USA | WB (1:1000) |
| DLL4 Antibody | CST (2589) , Boston, USA | WB (1:1000) |
| Jagged1 (28H8) Rabbit mAb | CST (2620) , Boston, USA | WB (1:1000) |
| Jagged2 (C23D2) Rabbit mAb | CST (2210) , Boston, USA | WB (1:1000) |
| ADAM17 (D22H4) Rabbit mAb | CST (6978) , Boston, USA | WB (1:1000) |
| Notch2 (D76A6) Rabbit mAb | CST (5732) , Boston, USA | WB (1:1000)  IP (1:50)  IHC (1:50)  IF (1:50) |
| Notch3 (D11B8) Rabbit mAb | CST (5276) , Boston, USA | WB (1:1000) |
| NOTCH4 (EPR18049) Rabbit mAb | Abcam (ab184742), Cambridge, UK | WB (1:1000) |
| Notch1 (EPR1238Y) Rabbit mAb | Abcam (ab52627), Cambridge, UK | WB (1:1000) |
| Histone H3 (D1H2) Rabbit mAb | CST (4499) , Boston, USA | WB (1:1000) |
| β-Catenin (D10A8) XP® Rabbit mAb | CST (8480) , Boston, USA | WB (1:1000)  IHC (1:50)  IF (1:50) |
| TCF4/TCF7L2 (C9B9) Rabbit mAb | CST (2565) , Boston, USA | WB (1:1000)  IF (1:50) |
| JUN Polyclonal antibody | proteintech (24909-1-AP), Wuhan, China | WB (1:1000) |
| c-Myc (D84C12) Rabbit mAb | CST (5605) , Boston, USA | WB (1:1000) |
| WNT3A Polyclonal antibody | proteintech (26744-1-AP), Wuhan, China | WB (1:1000) |
| LEF1 Polyclonal antibody | proteintech (14972-1-AP), Wuhan, China | WB (1:1000) |
| Ubiquitin Monoclonal antibody | HUABIO (ET1609-21), Hangzhou, China | WB (1:1000) |

Table S6. Premier sequences used in this study.

| Gene | Forward | Reverse |
| --- | --- | --- |
| JUN | TCCAAGTGCCGAAAAAGGAAG | CGAGTTCTGAGCTTTCAAGGT |
| JAG1 | GAGCAGTAGTTCCCCATT | GGGTTCCCAGGCACAGAA |
| ADAM10 | GCTCATTGGTGGGCAGTATTA | AAGTGCCTGGAAGTGGTTTAG |
| NOTCH2 | CTTCAGTGGTATGGACTGTGAG | GCAGAGGCAGGAGAAAGTATT |
| TCF7L2 | GCCATGGAGGTACAGACAAA | GCTGGTTTGGAGGAAGGATAG |
| MYC | CATACATCCTGTCCGTCCAAG | GAGTTCCGTAGCTGTTCAAGT |
| FOSL1 | CTCTGACCTACCCTCAGTACA | AGCCCAGATTTCTCATCTTCC |
| CCND1 | GCGGAGGAGAACAAACAGAT | GAGGGCGGATTGGAAATGA |
| CCND2 | TTCCCTCTGGCCATGAATTAC | GGGCTGGTCTCTTTGAGTTT |
| CCND3 | ACCTGGCTGCTGTGATTG | CGGGTACATGGCAAAGGTATAA |
| GAPDH | GGAAGGTGAAGGTCGGAGTC | GTTGAGGTCAATGAAGGGGTC |
| TCF7L2 promoter | CACTCCTCCCTCCGTCTCC | GAGGGAAAGAAGGAGGAAGAG |
